# Growth hormone secretagogues hexarelin and JMV2894 protect skeletal muscle from mitochondrial damages in a rat model of cisplatin-induced cachexia

**DOI:** 10.1038/s41598-017-13504-y

**Published:** 2017-10-12

**Authors:** Giuseppe Sirago, Elena Conte, Flavio Fracasso, Antonella Cormio, Jean-Alain Fehrentz, Jean Martinez, Clara Musicco, Giulia Maria Camerino, Adriano Fonzino, Laura Rizzi, Antonio Torsello, Angela Maria Serena Lezza, Antonella Liantonio, Palmiro Cantatore, Vito Pesce

**Affiliations:** 1 Department of Biosciences, Biotechnologies and Biopharmaceutics, University of Bari “A. Moro”, Bari, Italy; 2 Department of Pharmacy-Drug Sciences, University of Bari “A. Moro”, Bari, Italy; 3 Max Mousseron Institute of Biomolecules UMR5247, CNRS, University of Montpellier, ENSCM, Montpellier, France; 4 IBBE Institute of Biomembranes and Bioenergetics CNR-National Research Council of Italy, Bari, Italy; 5 School of Medicine and Surgery, University of Milano-Bicocca, Monza, Italy

## Abstract

Chemotherapy can cause cachexia, which consists of weight loss associated with muscle atrophy. The exact mechanisms underlying this skeletal muscle toxicity are largely unknown and co-therapies to attenuate chemotherapy-induced side effects are lacking. By using a rat model of cisplatin-induced cachexia, we here characterized the mitochondrial homeostasis in tibialis anterior cachectic muscle and evaluated the potential beneficial effects of the growth hormone secretagogues (GHS) hexarelin and JMV2894 in this setting. We found that cisplatin treatment caused a decrease in mitochondrial biogenesis (PGC-1α, NRF-1, TFAM, mtDNA, ND1), mitochondrial mass (Porin and Citrate synthase activity) and fusion index (MFN2, Drp1), together with changes in the expression of autophagy-related genes (AKT/FoxO pathway, Atg1, Beclin1, LC3AII, p62) and enhanced ROS production (PRX III, MnSOD). Importantly, JMV2894 and hexarelin are capable to antagonize this chemotherapy-induced mitochondrial dysfunction. Thus, our findings reveal a key-role played by mitochondria in the mechanism responsible for GHS beneficial effects in skeletal muscle, strongly indicating that targeting mitochondrial dysfunction might be a promising area of research in developing therapeutic strategies to prevent or limit muscle wasting in cachexia.

## Introduction

Cachexia is a severe syndrome characterized by loss of skeletal muscle and fat mass, often associated with cancer^1,2^. Cachexia is considered an index of a poor prognosis, having negative impact on the patients’ quality of life and survival. Muscle wasting in cachexia is due to increase of proteolysis and decrease of protein synthesis; it is mainly caused by activation of the pathways involving the ubiquitin (Ub)-proteasome, the mitogen-activated protein (MAP) kinases and myostatin systems^3^.

A multimodal approach with nutritional support, moderate exercise and treatment with corticosteroids and progestational drugs is currently proposed for cachectic syndromes^4,5^. However, these treatments have not proven to be clinically effective and therefore there is a pressing need for newer clinical approaches to cachexia. Ghrelin is a 28-amino acid peptide produced by the oxyntic cells of the stomach, and it is a natural ligand for the growth hormone (GH)-secretagogue receptor (GHS-R-1a). Based on its ability to stimulate appetite, lean and fat mass, ghrelin has been proposed as a therapeutic option for the treatment of cachexia^6^. However, the clinical use of this drug has been limited because of its short half-life and low plasma stability^7,8^. For these reasons, several synthetic growth hormone secretagogues (GHS) have been developed and are now studied for cachexia treatment^9–11^.

A better knowledge of the mechanisms of action of GHS in cachexia is needed, since GHS compounds are presently under consideration by the European Medicine Agency for approval to be introduced into clinical practice for treatment of cachexia^12^.

Cisplatin, a cytotoxic agent widely used in cancer treatment, also induces cachexia. Cisplatin-treated animals are a suitable animal model to study muscle derangements specifically associated with chemotherapy^13,14^ and can be used for testing new therapies for cachexia^6,15–18^. Using a rat model of cisplatin-induced cachexia, we recently demonstrated that, consistently with the loss of skeletal muscle mass and occurrence of muscle weakness, the extensor digitorum longus (EDL) muscle of cachectic rats is characterized by a disrupted sarcoplasmic reticulum (SR) calcium signaling and an alteration of sarcolemma excitability^18^. Furthermore, according to the multiple actions proposed for ghrelin and GHS^11^, we demonstrated that administration of JMV2894, a novel peptidomimetic GHS, and hexarelin, a well-known synthetic hexapeptide, antagonized cisplatin-induced muscle wasting. This was obtained by efficaciously preventing calcium homeostasis dysregulation both in terms of calcium movements and gene expression regulation of specific calcium-related proteins^18^.

Muscle wasting in cachectic patients is likely multifactorial and the underlying mechanisms remain largely unknown. Mitochondria are very important for efficient skeletal muscle functionality^19–21^, since they are involved in redox homeostasis and calcium signaling^22^. Muscle fiber degradation that occurs during clinical and experimental cancer cachexia is related to an alteration of mitochondrial structure and function^21,23,24^ as well as to alterations of calcium homeostasis^24,25^. Only recently the involvement of mitochondrial dysfunction in chemotherapy-induced cachexia has been described^13,26^.

Following these lines of evidence, we investigated the association between the development of cisplatin-induced cachexia and the alteration of mitochondrial function, evaluating the effects of JMV2894 and hexarelin treatment. In particular, by assessing several mitochondrial biomarkers in tibialis anterior (TA) skeletal muscle, we explored mitochondrial biogenesis and dynamics, mitochondrial oxidative stress balance and autophagy pathways associated with the cachectic muscle. Our results show that cisplatin treatment causes severe alterations of the mitochondrial homeostasis and that JMV2894 and hexarelin are capable to antagonize chemotherapy-induced impairment of mitochondrial function. They also indicate a key-role played by mitochondria in the mechanism responsible for the GHS beneficial effects in skeletal muscle, further corroborating the current hypothesis^27^ that targeting mitochondrial dysfunction might be a promising area of research to develop therapeutic strategies for preventing or limiting muscle wasting.

## Results

### Effect of cisplatin and GHS treatment on body and muscle weight

Cisplatin induced a significant decrease in body weight compared to control rats, whereas GHS administration partially opposed this change (Table 1). The reduction in body weight was associated with a significant decrease in cumulative food intake in cisplatin-treated rats, which was partially antagonized by GHS administration^18^.

**Table 1:** Effect of cisplatin and GHSs on the body and tissue weight of the animals

	Control (n = 4)	Cisplatin (n = 4)	Cisplatin + Hexarelin (n = 4)	Cisplatin + JMV2894 (n = 4)
**Body weight** **(g)**				
initial (day 0)	338 ± 14	341 ± 13	340 ± 24	342 ± 2.0
final (day 5)	362 ± 13	317 ± 4.8	348 ± 25	334 ± 7.0
	(+7.1%)	(−7.0%)	(+2.4%)	(−2.4%)
**Muscle (TA) weight**				
(mg/g total body)	2.30 ± 0.14	1.96 ± 0.02*	2.27 ± 0.07^#^	2.20 ± 0.06^§^
**Heart weight**				
(mg/g total body)	3.46 ± 0.30	3.41 ± 0.30	3.31 ± 0.23	3.33 ± 0.20
**Kidney weight**				
(mg/g total body)	3.61 ± 0.29	3.50 ± 0.18	3.50 ± 0.21	3.62 ± 0.10

TA, Tibialis anterior. The numbers in parentheses indicate the percent variation of the body weight between days 5 and 0.

*p < 0.05 (cisplatin/control comparison); ^#^p < 0.05 (cisplatin + hexarelin/cisplatin comparison); ^§^p < 0.05 (cisplatin + JMV2894/cisplatin comparison).

Cisplatin caused a significant decrease (about 15%) in the muscle/body weight ratio compared to control rats (Table 1). This change was completely inhibited by the administration of both GHS. Similar results, concerning the EDL muscle, were recently obtained^18^. No significant alterations were observed in heart and kidney weight in all treatment groups. These results show that cisplatin induces atrophy in rat TA muscle and that this effect is antagonized by the treatment with each of the two GHS.

### Mitochondrial biogenesis in cisplatin-treated rats and effects of GHS

**Figure 1:**
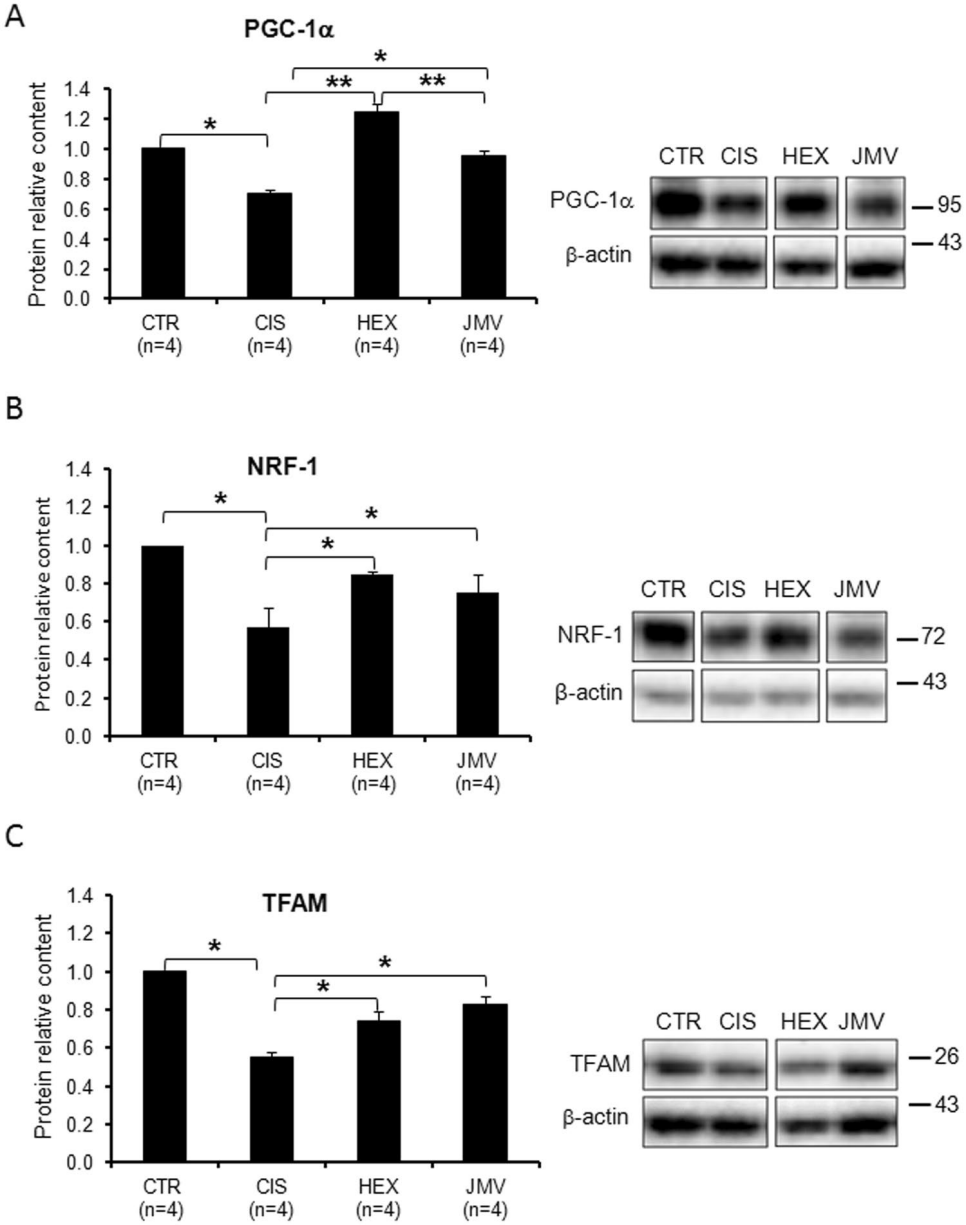
Effect of cisplatin and GHS treatments on the level of factors regulating the mitochondrial biogenesis. (**A**) The histogram shows the relative content of PGC-1α protein normalized with respect to β-actin content. Cisplatin treatment decreases the level of the protein; the administration of hexarelin and, to a lesser extent of JMV, tends to restore the control value. (**B**) The histogram shows the relative content of NRF-1 protein normalized with respect to β-actin content. Cisplatin treatment decreases the level of the protein; the administration of hexarelin and JMV tends to restore the control value. (**C**) The histogram shows the relative content of TFAM normalized with respect to β-actin. Cisplatin treatment lowers the level of TFAM; the administration of the two segretagogues brings the protein level near to that of controls. The reported results are the average (±SEM) of experiments performed in quadruplicate. CTR, controls; CIS, rats treated with cisplatin; HEX, rats treated with cisplatin and hexarelin; JMV, rats treated with cisplatin and JMV2894. All data are normalized to control samples set as 1. n, number of animals of each group. *p < 0.05; **p < 0.01. A representative western blot is also shown. White spaces between blots indicate not adjacent lanes deriving from different parts of the same gel. The whole blot is reported in Supplementary Figure S2. The position of molecular weight markers is reported at the right of each blot.

**Figure 2:**
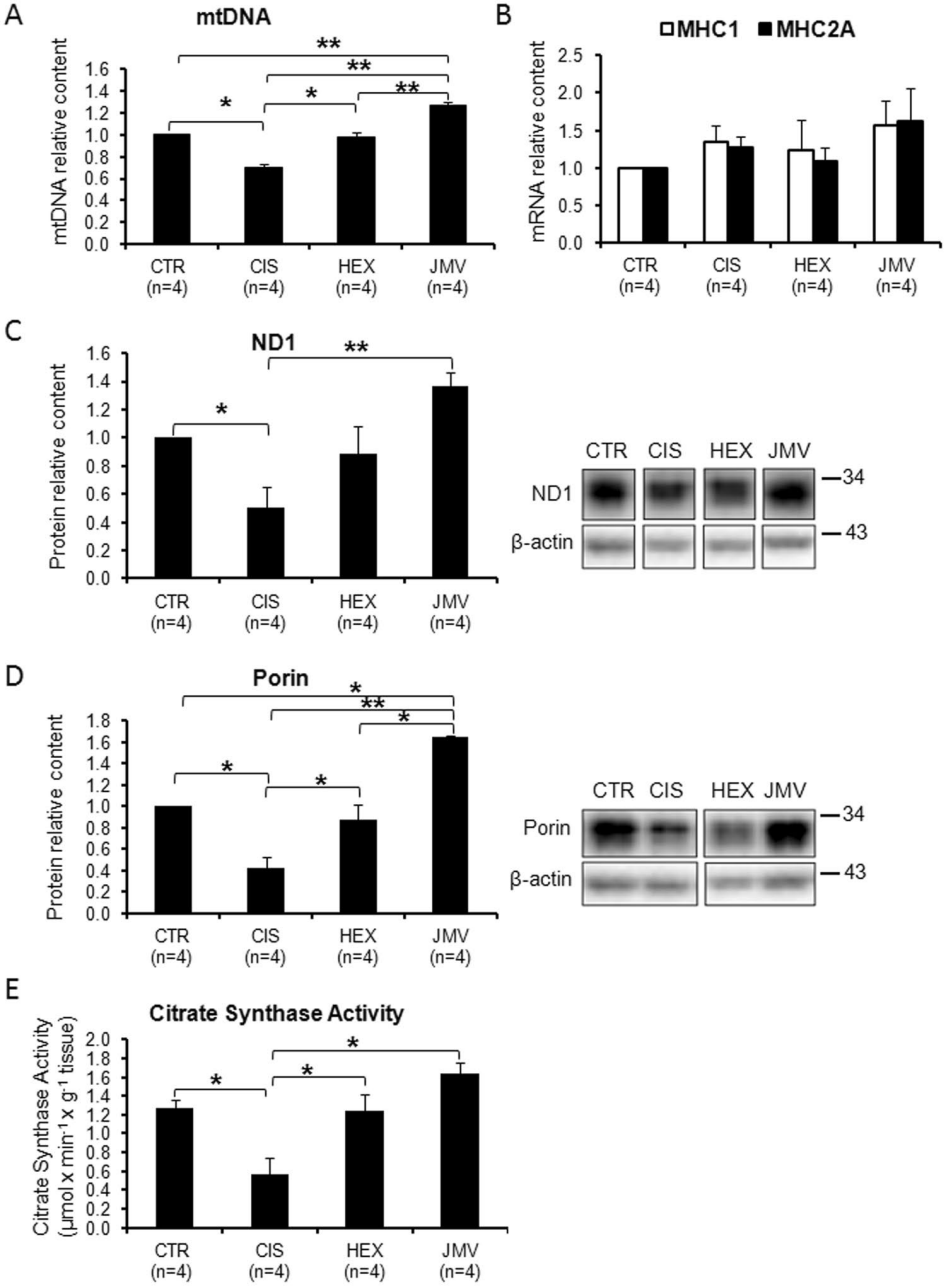
Changes of mitochondrial biogenesis and mitochondrial mass following cisplatin and GHS treatments. (**A**) MtDNA copy number. The mtDNA copy number decreases in the rats treated with cisplatin, while the administration of hexarelin and JMV brings the level to that of control samples. (**B**) Content of MHC1 and MHC2A mRNAs. The histograms show the relative content of the two mRNAs normalized with respect to β-actin mRNA. No significant changes following cisplatin and GHS treatments were observed. (**C**) ND1 content. The histogram shows the relative content of ND1 normalized with respect to β-actin. Cisplatin treatment causes a decrease of ND1 while the administration of hexarelin and at a higher extent of JMV2894 prevents such decrease. (**D**) Porin content. The histogram shows the relative content of Porin normalized with respect to β-actin. Cisplatin treatment causes a decrease of Porin and that the administration of hexarelin and at a higher extent of JMV2894 prevents such decrease. (**E**) Citrate Synthase activity. The histogram shows that cisplatin treatment causes a decrease of citrate synthase and that the administration of hexarelin and JMV prevents such decrease. The reported results are the average (±SEM) of experiments performed in quadruplicate. CTR, controls; CIS, rats treated with cisplatin; HEX, rats treated with cisplatin and hexarelin; JMV, rats treated with cisplatin and JMV2894. All data are normalized to control samples. n, number of animals of each group. *p < 0.05; **p < 0.01). For the proteins a representative western blot is also shown. White spaces between blots indicate not adjacent lanes deriving from different parts of the same gel. The whole blot is reported in Supplementary Figure S2. The position of molecular weight markers is reported at the right of each blot.

To evaluate the effect of cisplatin on mitochondrial biogenesis, we measured the content of three proteins involved in mtDNA replication and maintenance, i.e. the Peroxisome proliferator-activated receptor γ Coactivator-1α (PGC-1α), the Nuclear Respiratory Factor 1 (NRF-1) and the Mitochondrial Transcription Factor A (TFAM). PGC-1α is a nuclear coactivator functioning as a master regulator of mitochondrial biogenesis and activates, among others, the expression of NRF-1. NRF-1 is a nuclear transcription factor that activates the expression of many mitochondrial genes, included TFAM. This is a mitochondrial protein controlling both mtDNA maintenance and replication as well as mitochondrial transcription^28–30^. The content of PGC-1α, NRF-1 and TFAM decreased in rats treated with cisplatin to an extent ranging from 30% to 50% compared to control (Fig. 1A–C). Treatment with hexarelin and JMV2894 prevented the reduction of the level of the three proteins, with hexarelin particularly effective on PGC-1α. Considering the strong link between TFAM and mtDNA transcription and replication^31^ we also analyzed the mtDNA content. Changes in mtDNA level were in agreement with those of the proteins: Fig. 2A shows that in cisplatin-induced cachectic rats, mtDNA decreased about 30% compared to controls and that this change was fully antagonized by both GHS, with JMV2894 showing an higher effect. To determine whether this GHS-induced mtDNA increase was associated with a fiber switch toward a more oxidative phenotype in the fast TA muscle, the expression of the transcripts of the oxidative isoforms of myosin heavy chain (MHC), type 1 and type 2a, was measured by quantitative real-time PCR. As shown in Fig. 2B, the gene expression of *Myh7*, encoding for MHC1, and of *Myh2*, encoding for MHC2a, did not change significantly among the four animals groups, thus indicating that the changes induced by GHS in mtDNA were not associated with a fiber type switch.

The changes on mitochondrial biogenesis were further evaluated by analyzing the level of ND1, a mtDNA coded subunit of respiratory Complex I. Figure 2C shows a behavior similar to mtDNA: in this case JMV2894 was particularly effective in increasing the level of the protein with respect to hexarelin. We also measured the effect of treatments on mitochondrial mass by evaluating the content of porin and the activity of citrate synthase (CS), an enzyme marker of mitochondrial mass in physiological and pathological conditions^32^. The two markers behaved in a similar way: cisplatin remarkably reduced porin content and CS activity (about 50%) and both GHS effectively antagonized these decreases with JMV2894 displaying a stronger effect (Fig. 2D,E).

The overall outcome of these experiments is that cisplatin treatment induced a decline of mitochondrial biogenesis and mitochondrial mass in rat TA muscle and that both hexarelin and JMV2894 antagonized cisplatin effects.

### Mitochondrial Oxidative stress in cisplatin-treated rats and effects of GHS

Reactive oxygen species (ROS) production increases in various physiopathological conditions such as in muscle atrophy^33–36^. Here, we evaluated the effect of cisplatin and GHS treatments on oxidative stress by measuring in TA muscle the level of three proteins involved in ROS metabolism: i) cellular overoxidized peroxiredoxins (PRX-SO_3_); ii) peroxiredoxin III, (PRX III); iii) mitochondrial superoxide dismutase (MnSOD). PRXs proteins are involved in a scavenger cycle that is used to remove ROS species from cytosol, and especially from mitochondria that are the main source of ROS. When the scavenger cycle is unbalanced and ROS presence is high, there is an increase in overoxidized PRXs, suggesting an increased oxidative stress^37^. On the other hand the activation of ROS detoxification mechanisms is associated with the increase of PRX III and MnSOD^38,39^.

**Figure 3:**
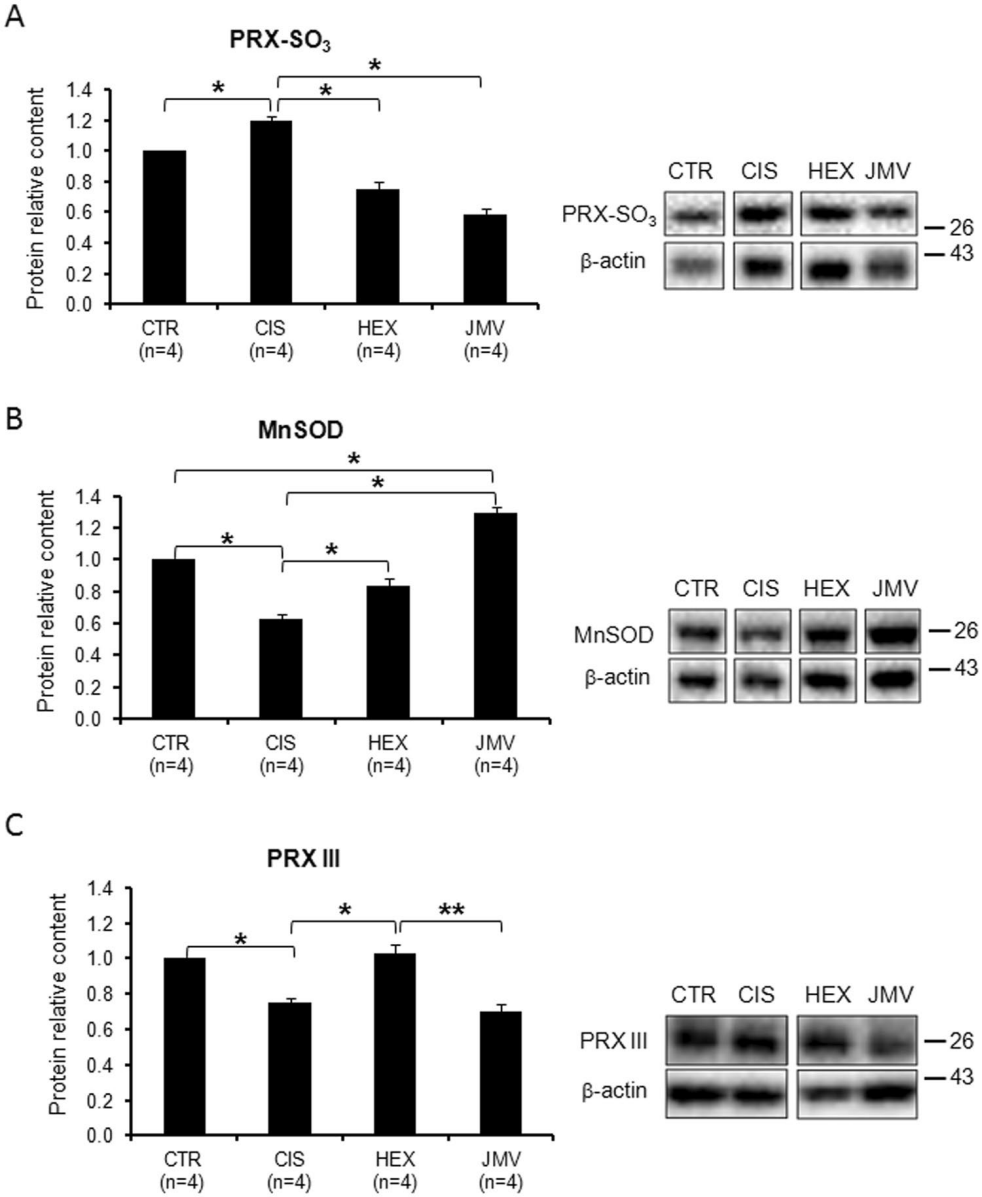
Mitochondrial oxidative stress in cisplatin-treated rats and after GHS administration. (**A**) The relative content of overoxidized PRXs (PRX-SO_3_) is higher in the cisplatin-treated rats; hexarelin and JMV treatment prevent such increase. (**B**) The relative content of MnSOD is lower in the cisplatin-treated rats; the two segretagogues prevent the decrease. (**C**) The relative content of PRX III is lower in the cisplatin-treated rats; hexarelin administration prevents the decrease while JMV does not. The reported results are the average ( ± SEM) of experiments performed in quadruplicate. All data are normalized to control samples. n, number of animals of each group. *p < 0.05; **p < 0.01. CTR, controls; CIS, rats treated with cisplatin; HEX, rats treated with cisplatin and hexarelin; JMV, rats treated with cisplatin and JMV2894. A representative western blot is also shown. White spaces between blots indicate not adjacent lanes deriving from different parts of the same gel. The whole blot is reported in Supplementary Figure S2. The position of molecular weight markers is reported at the right of each blot.

Figure 3(A–C) shows that cisplatin treatment enhanced the oxidative stress as indicated by the increase in PRX-SO_3_ proteins level, associated with the decrease in PRX III and MnSOD. Both hexarelin and JMV2894 prevented this trend restoring the MnSOD and PRX III proteins content. In particular, while hexarelin treatment restored the content of both proteins near to the control value, JMV2894 rescued MnSOD level even above the value of control animals, but failed to prevent the cisplatin-induced PRX III reduction. This result could suggest that the lower efficiency of JMV2894 on PRX III level is partially compensated by its higher efficiency in stimulating MnSOD level.

### Mitochondrial dynamics in cisplatin-treated rats and effects of GHS

**Figure 4:**
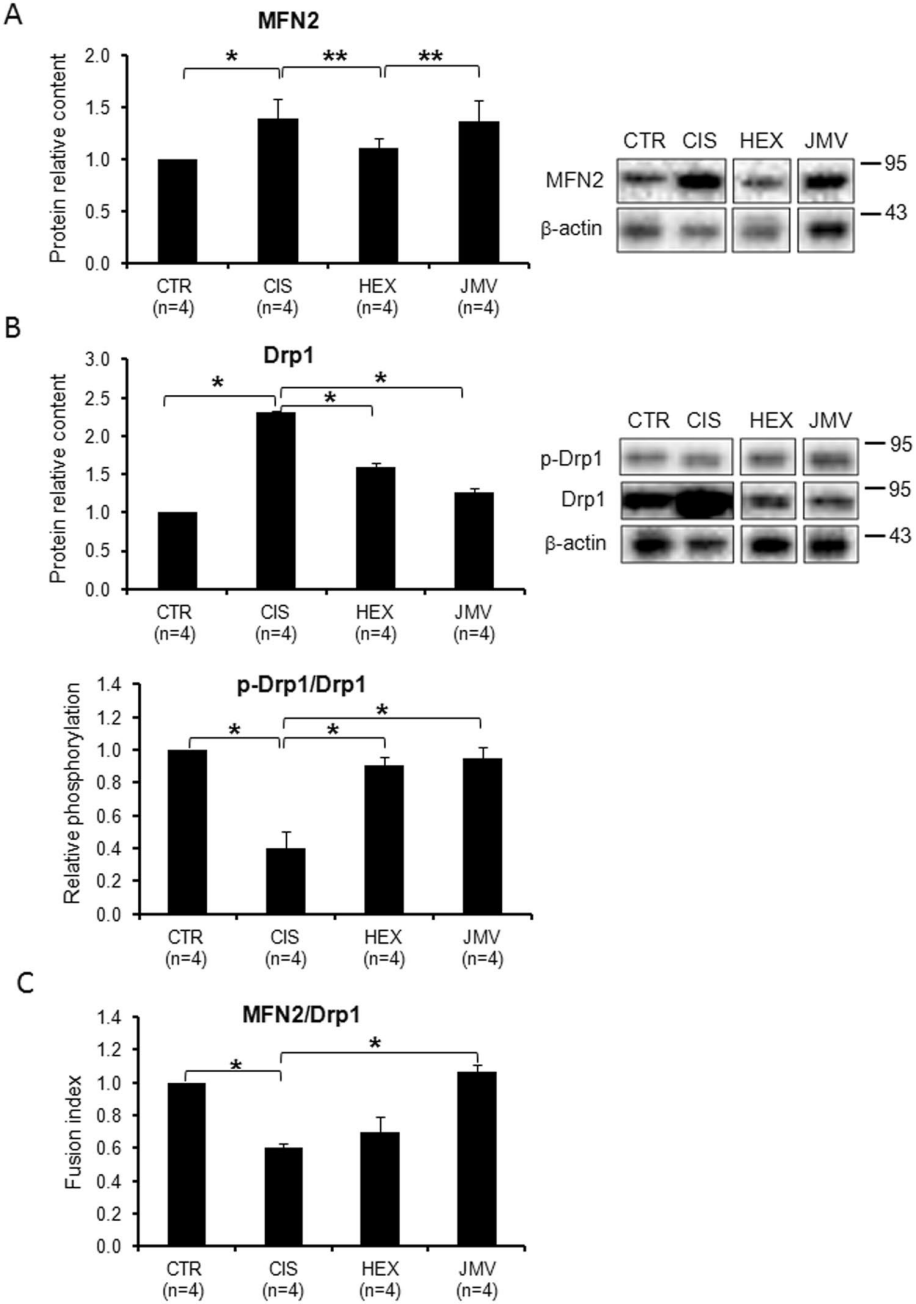
Mitochondrial dynamics in cisplatin-treated rats and after GHS administration. (**A**) The relative content of MFN2 increases in cisplatin-treated rats; treatment with hexarelin, but not with JMV2894, prevented such increase. (**B** top part) Drp1 increases in cisplatin-treated rats; the administration of the two GHS tends to lower such increase. (**B** bottom part) Cisplatin induced a decrease of the phosphorylation of Drp1 at S637 residue, whereas GHS prevent such changes. (**C**) The ratio between MFN2 and Drp1 (Fusion Index) decreases in cisplatin-treated rats. Hexarelin and more effectively JMV2894 administration tend to bring back the value of the ratio to that of control. Results are the average (mean ± SEM) of experiments performed in quadruplicate. All data have been normalized to control samples. n, number of animals of each group. *p < 0.05; **p < 0.01. A representative western blot is shown (CTR, controls; CIS, rats treated with cisplatin; HEX, rats treated with cisplatin and hexarelin; JMV, rats treated with cisplatin and JMV2894). White spaces between blots indicate not adjacent lanes deriving from different parts of the same gel. The whole blot is reported in Supplementary Figure S2. The position of molecular weight markers is reported at the right of each blot.

The regulation of mitochondrial dynamics (fission and fusion) is critical for mitochondrial function, morphology and distribution and plays a key-role in mitochondrial turnover and life cycle in muscle^21^. Since abnormal mitochondria have been observed in cachectic muscles^40^ we evaluated changes in mitochondrial dynamics in cisplatin-induced cachexia and after GHS administration. We measured, in the rat TA skeletal muscle, the level of two key proteins involved in these processes, namely dynamin-related protein 1 (Drp1), which regulates mitochondrial fission, and mitofusin 2 (MFN2), the most abundant mitofusin isoform in muscle^21^, implicated in mitochondrial fusion. In cisplatin-treated group, the level of both MFN2 and Drp1 increased compared to control rats (Fig. 4A and top part of 4B).The treatment with the two GHS induced different effects: Drp1 level decreased and reached a value close to control after treatment with hexarelin or JMV2894, whereas only hexarelin was able to effectively restore the protein level of MFN2 at control value.

Since it has been reported that Drp1 activity is linked to its translocation to mitochondria and that phosphorylation of Drp1 at serine 637(S637) suppresses its mitochondrial translocation and inhibits Drp1 GTPase activity^41^, we tested the changes in the phosphorylation state of Drp1 at S637 in our experimental model. Figure 4B (bottom part) shows a decreased phosphorylation in cisplatin-treated rats and that the treatment with both GHS prevented such change. This result indicates that cisplatin not only increases the Drp1 level, but also stimulates its fission activity.

We evaluated also the Fusion Index (FI) calculated as MFN2/Drp1 ratio, which measures the ratio between mitochondrial fusion and fission. In the cisplatin-treated group we observed a decrease in the FI (Fig. 4C) suggesting fission as the prevailing process occurring in the cachectic animal model. Hexarelin and JMV2894 both stimulated a recovery of FI that was higher in JMV2894-treated animals.

### Autophagy in cisplatin-treated rats and effects of GHS

It is well known^42^ that the phosphorylation state of AKT controls the phosphorylation and consequently the localization of the Forkhead boxO3a (FoxO3a) factor: when AKT is active (phosphorylated) it phosphorylates FoxO3a which remains inactive in the cytoplasm. On the contrary when AKT is inactive, FoxO3a is dephosphorylated and migrates to the nucleus where it activates genes, such as Atg1 and Murf1 that promote muscle wasting. To obtain information on the role of this pathway in our experimental system, we tested the phosphorylation state of its components upon cisplatin and GHS treatments.

**Figure 5:**
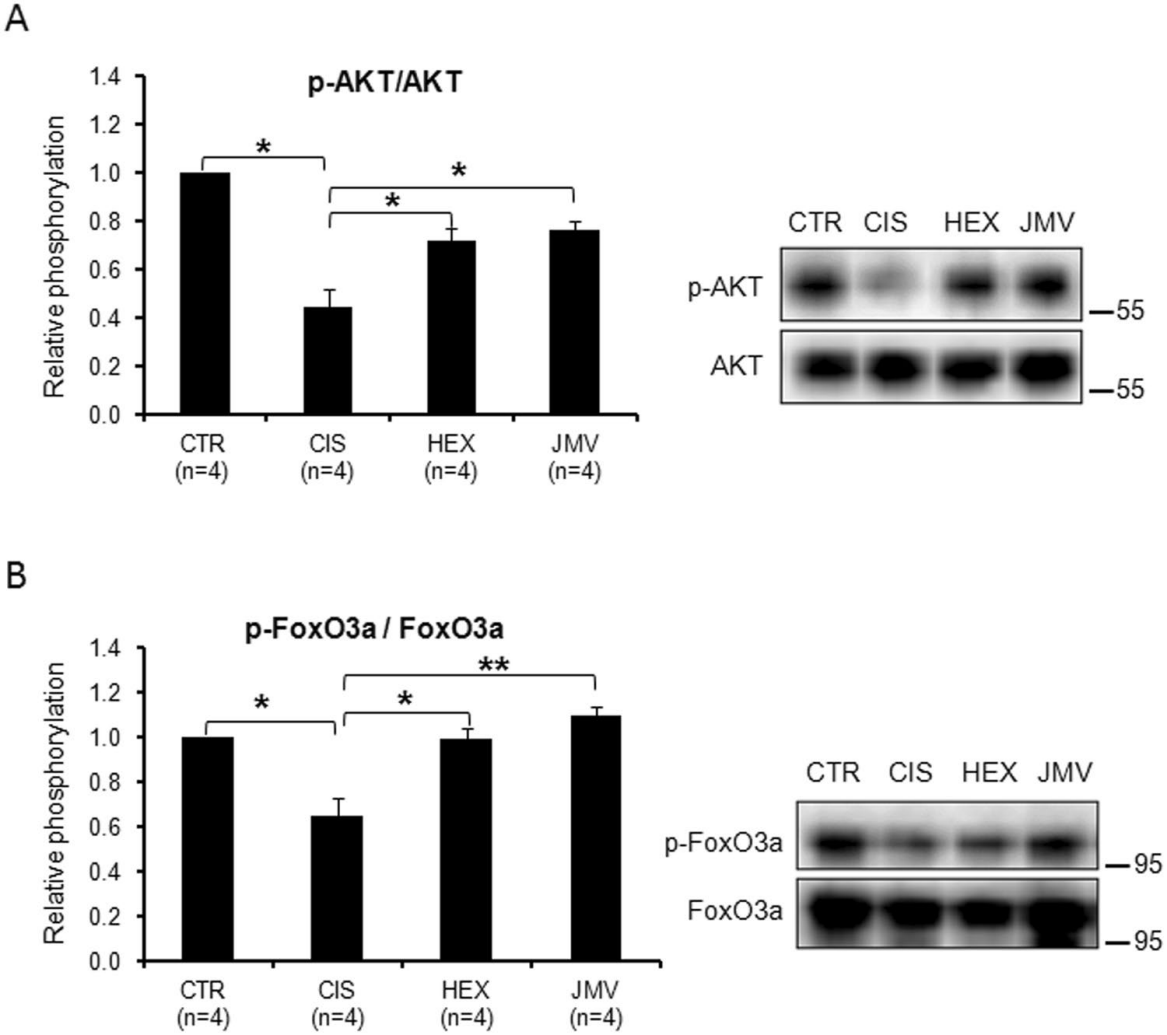
Phosphorylation level of AKT and FoxO3a in cisplatin-treated rats and after GHS administration. (**A**) The AKT phosphorylation level decreases in cisplatin-treated rats and increases after the administration of the two segretagogues. (**B**) FoxO3a phosphorylation decreases in cisplatin-treated rats and increases after the administration of the two segretagogues. Results are the average (mean ± SEM) of experiments performed in quadruplicate. All data have been normalized to control samples. n, number of animals of each group. *p < 0.05; **p < 0.01. CTR, controls; CIS, rats treated with cisplatin; HEX, rats treated with cisplatin and hexarelin; JMV, rats treated with cisplatin and JMV2894. A representative western blot is also shown. The whole blot is reported in Supplementary Figure S2. The position of molecular weight markers is reported at the right of each blot.

Figure 5A shows that the phospho-AKT to total AKT ratio was significantly reduced in cisplatin-treated rats compared to control animals, thus indicating a reduced presence of the active protein in cachectic TA muscle. The reduction of AKT phosphorylating activity should lead to a diminished phosphorylation of FoxO3a and favour its localization in the nucleus where it activates autophagy genes. This prevision was confirmed by the data reported in Fig. 5B where it appears that cisplatin treatment caused a decrease in the level of phosphorylated FoxO3a compared to control rats. The administration of hexarelin or JMV2894 prevented such changes inducing a recovery of the phosphorylated AKT and FoxO3a forms. This should favour FoxO3a localization in the cytoplasm and lead to its inactivation. These results indicate that cisplatin treatment had a detrimental effect on the phosphorylation of AKT and FoxO3a and that GHS treatment opposed these effects.

**Figure 6:**
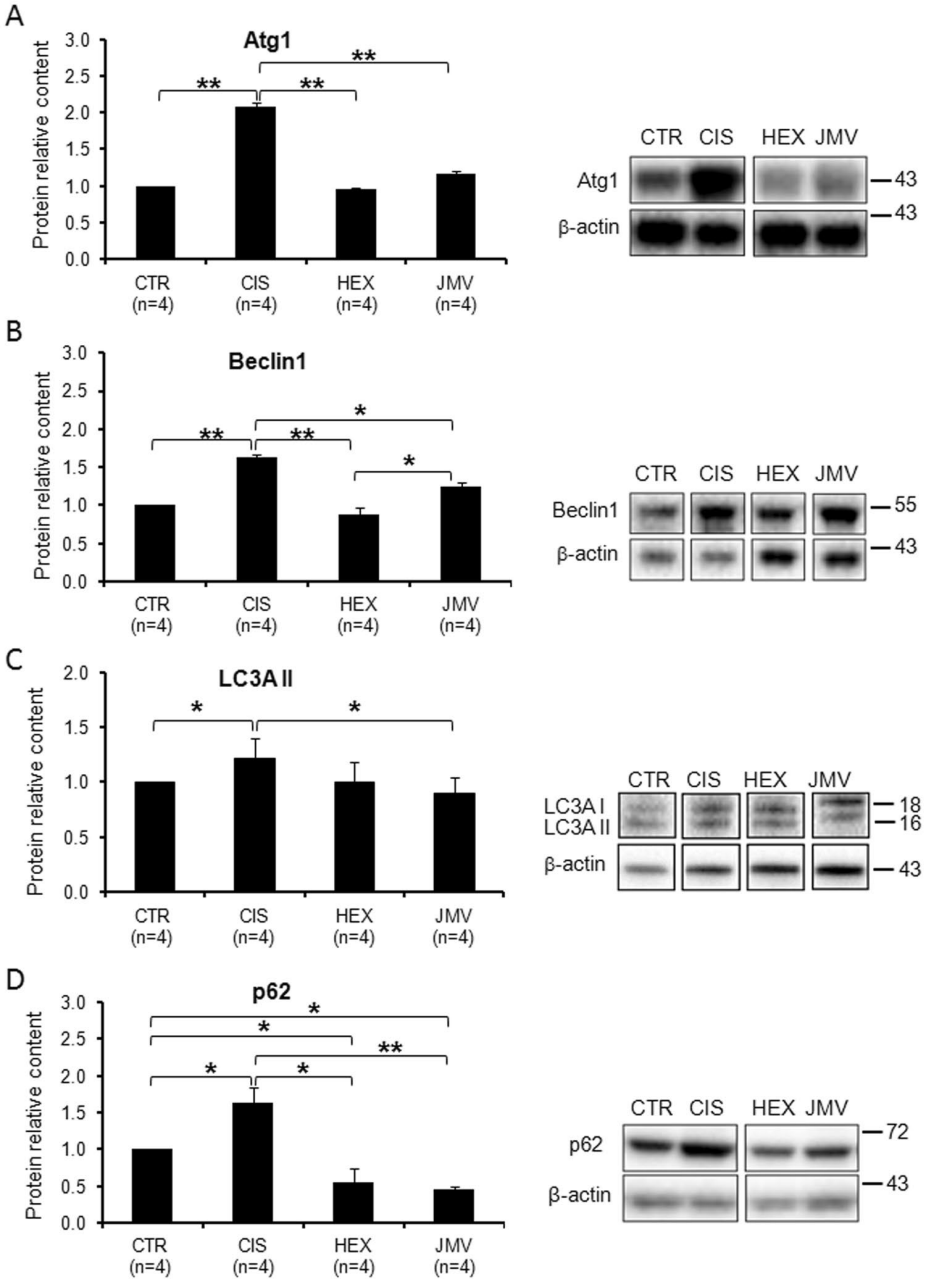
Changes in the level of autophagy marker proteins in cisplatin-trated rats and after GHS administration. (**A**) Atg1 increases in the cisplatin-treated rats. The administration of hexarelin or JMV2894 prevents such a change. (**B**) Beclin1 content increases in cisplatin groups; hexarelin and JMV2894 treatments bring back the value to that of control samples. (**C**) LC3AII slightly increases in the cisplatin-treated rats. JMV2894 treatment prevents such a change. (**D**) p62 increases in the cisplatin-treated rats. The administration of hexarelin or JMV2894 prevents such a change leading to a value lower than control. Results are the average (mean ± SEM) of experiments performed in quadruplicate. All data have been normalized to control samples. n, number of animals of each group. *p < 0.05; **p < 0.01. CTR, controls; CIS, rats treated with cisplatin; HEX, rats treated with cisplatin and hexarelin; JMV, rats treated with cisplatin and JMV2894. A representative western blot is also shown. White spaces between blots indicate not adjacent lanes deriving from different parts of the same gel. The whole blot is reported in Supplementary Figure S2. The position of molecular weight markers is reported at the right of each blot.

The AKT/FoxO3a axis controls also the expression of the gene for Atrogin 1 (Atg1); this protein, also called Muscle atrophy F-box (MAFbx), is an E3 ubiquitin ligase, abundant in skeletal muscle, which initiates ATP-dependent ubiquitin-mediated proteolysis and promotes muscle atrophy^43^. In cisplatin-treated rats, we observed a strong increase in Atg1 protein level (Fig. 6A), which suggests an increase of protein degradation as confirmed by the loss of weight of the TA muscle. Consistently with the effect on the phosphorylated FoxO3a protein level, both GHS efficaciously rescued the altered expression of Atg1.

The evaluation of the protein content of Beclin1, p62 and of LC3AII, the lipidated form of LC3, was then used as a measure of autophagy^44^. Figure 6B shows that the content of Beclin1, a protein involved in the starting of autophagy, increased significantly in cisplatin-treated rats suggesting autophagy activation. Hexarelin, and to a smaller extent JMV2894, prevented the protein increase. We then evaluated the level of the lipidated form of LC3 (LC3AII), an ubiquitin-like molecule essential for the formation of the autophagosome. Figure 6C shows that in cisplatin-treated rats the content of LC3AII protein increased slightly, while JMV2894 treatment prevents such a change. Finally we measured the content of p62, an adaptor molecule that targets protein aggregates to the autophagosome binding simultaneously LC3 and the ubiquitinated proteins. p62 is degraded during autophagy and therefore its level should decrease when autophagy is induced. Surprisingly, we found that p62 increased in cisplatin treated rats, whereas GHS treatments decreased considerably the protein level to a value lower than controls (Fig. 6D).

We can interpret these data assuming that in cisplatin-treated rats, the increase of Beclin1 and the LC3AII indicate induction of autophagy. On the other hand the increase of p62 suggests some problems in this process, perhaps due to impairments in autophagosome clearance. Interestingly the treatment with both GHS exert a protective effect preventing such phenomena.

## Discussion

Currently pharmacological treatments of cachexia, including administration of progestational drugs and corticosteroids, are not satisfactorily effective and a better understanding of this condition could lead to development of new and more effective drugs. Here we used an animal model of cisplatin-induced cachexia to gain insights into the role of mitochondria in chemotherapy-related muscle wasting, and highlighted the effects of hexarelin and JMV2894 treatment as a possible therapeutic approach.

### Characterization of cisplatin-induced muscle mitochondrial damage

The first aim of this study was to characterize the effect of cisplatin on the oxidative metabolism in rat TA muscle. We found that cisplatin treatment caused a reduction of muscle weight and a decline of several parameters linked to mitochondrial function. In particular, cisplatin induced a decrease in mitochondrial biogenesis and mitochondrial mass, an alteration of mitochondrial dynamics, increased expression of autophagy-related genes and enhanced ROS production. Similar mitochondrial dysfunctions and mitochondrial morphological abnormalities were reported in cancer cachexia^3^.

Among the alterations of the above reported parameters, change of PGC-1α seems to play a key role. Its decrease, observed in our cachectic rat model, might be due to the impairment of the PI3K-AKT-mTOR-YY1 signaling pathway. Such hypothesis is supported by the reduction of the AKT phosphorylation that occurs in cisplatin-treated rats. PGC-1α is a transcriptional coactivator involved in the formation/maintenance of slow-twitch fibers in skeletal myocytes^45^. Moreover it has a major role in regulating mitochondrial biogenesis through the activation of nuclear respiratory factors^28^ (NRFs) that promote the expression of different nuclear-encoded mitochondrial proteins. These include TFAM, which is mainly involved in mtDNA replication and transcription. Indeed cisplatin-treated rats exhibited reduced NRF-1, TFAM and mtDNA content as well as a reduction in ND1 and in mitochondrial mass. The decrease in mtDNA might also be a direct effect of cisplatin on mtDNA replication. It was reported that cisplatin accumulated in mitochondria^46^ and caused a direct and significant impairment of mtDNA and mtRNA synthesis and decreased steady-state levels of mtRNAs in rat liver mitochondria^47^. A decreased mtDNA content was reported^48^ in a cancer-cachexia model. Moreover Pesce *et al*.^49^ showed that the reduced availability of mtDNA is a limiting factor in mitochondrial gene expression in the skeletal muscle of aged rats.

PGC-1α controls the expression of detoxifying genes such as MnSOD and PRX III, therefore the decrease of PGC-1α is in agreement with the decrease of MnSOD and PRX III, which remove ROS species from the mitochondrial matrix. This reduced ROS scavenger capacity was confirmed by the increased amount of total overoxidized acidic PRXs isoforms (PRX-SO_3_), which correspond to the sulfinic and irreversibly inactivated sulfonic forms of the antioxidant enzymes^50^, likely caused by ROS accumulation in mitochondria. The diminished level of factors involved in mitochondrial biogenesis together with the reduced mtDNA content and a partial loss of mitochondrial antioxidant defense system could compromise the skeletal muscle fiber composition and ultimately the skeletal muscle performance in our experimental cachectic rat model.

PGC-1α plays also a crucial role in mitochondrial dynamics and in the regulation of mitochondrial structure and function during muscle atrophy^21^. In cisplatin-treated rats, we found an increase of both fusion (MFN2) and fission (Drp1) proteins, with a prevalence of fission compared to fusion, as suggested by the Fusion Index reduction. Moreover the phosphorylation state of Drp1 at S637 was decreased indicating an enhancement of the fission activity of the protein. As reported for cancer cachexia^24,48^, the cisplatin-induced alteration could lead to mitochondrial fragmentation, which is typically associated with atrophying muscles in various metabolic disorders. It is well known that in the skeletal muscle there is a bidirectional SR-mitochondria communication. On one hand Ca^2+^ released from SR stimulates mitochondrial ATP production, allowing to meet increased energy demand during excitation-contraction (EC) coupling, leading to muscle contraction. On the other hand, mitochondria can inhibit undesired calcium release from SR by controlling the local redox environment around the calcium release unit (CRU)^24,51,52^. Since mitochondrial Ca^2+^ signaling depends on interactions between mitochondria and ER/SR, via formation of MFN2 complexes, it is conceivable that alteration of MFN2 protein level can affect Ca^2+^ signaling. Thus the Ca^2+^ dysregulation observed in the same cachectic animal model^18^, can be related with the here reported increased expression of MFN2 and the occurrence of muscle weakness that we observed *in vivo* after cisplatin administration^18^ may result from a combined effect of mitochondria and SR dysfunctions.

Compared with control muscles, cachectic muscle in cisplatin-treated rats showed also a marked decrease in dephosphorylated AKT and dephosphorylated FoxO3a content associated with a significant increase of Atg1, of the autophagic markers Beclin1 and of LC3AII.

On the other hand we found an increase of p62, a protein that should be degraded during the autophagic process^53^. While the increase of Beclin1, and at a lower extent of LC3AII, may indicate an induction of autophagy, the increase of p62 can be ascribed to an autophagic defect, possibly due to the exhaustion of the lysosomal degradative capacity. This can lead to autophagosomal accumulation, dysfunction in cellular trafficking and abnormality in cytoskeleton organization, thus contributing to muscle atrophy^54^. The here observed loss of TA muscle weight supports this hypothesis. Increase of Beclin1 and p62, have been reported in the skeletal muscle of cachetic cancer patients and in muscle atrophy induced by cancer^55,56^. Moreover it has been shown that defective autophagy contributes to the pathogenesis of different forms of muscular distrophies associated with accumulation of altered organelles into myofibers or abnormal degradation of myofibers components^57^. Also ageing is accompanied by autophagic defects as shown by studies performed in human and mice skeletal muscles^58,59^. Overall these evidences support the concept that cisplatin treatment recapitulates the main features of cancer-induced cachexia, and thus making the cisplatin-treated rats an useful and valuable model to investigate the molecular bases of this syndrome.

### Effects of GHS administration in preventing cisplatin-induced muscle mitochondrial damage

The changes in mitochondrial metabolism due to cisplatin treatment were largely antagonized by the administration of hexarelin and JMV2894. This was shown by the attenuation in muscle weight loss and by restoration of parameters related to mitochondrial biogenesis and mass, mitochondrial dynamics, autophagy and oxidative stress. The non peptidic agonist JMV2894, was often more effective than the peptidic hexarelin in preventing cisplatin-induced alterations of cell metabolism. Similarly, JMV2894 was more effective in restoring calcium homeostasis and the expression of some calcium-related proteins^18,60^.

Our data are in agreement with previous reports showing that ghrelin administration prevented tumor and cisplatin-induced muscle wasting, through the down-regulation of inflammation of the p38/c/EBP-beta/myostatin axis and the activation of Akt, myogenin and myoD^3^. Ghrelin administration has been also shown to increase PGC-1α, to attenuate ROS production and to decrease apoptosis^6^. Moreover, ghrelin could have also an epigenetic effect as Tamaki *et al*.^61^ reported a demethylation of the cytosine residue upstream of the transcription initiation site of PGC-1α. It would be interesting to investigate the consequences of these effects and if these are extended to hexarelin and JMV2894.

Recently anamorelin a novel ghrelin-receptor agonist has been used for cachexia treatment. Clinical trials showed that in cachectic patients anamorelin administration significantly improved lean body mass, body weight and anorexia-cachexia-related symptom burden. However, handgrip strength, a measure of muscle function, did not significantly improve in the anamorelin group compared with placebo^12^. Interestingly, both hexarelin and JMV2894 partially prevented the muscle force reduction induced by cisplatin administration in rats^18^. All our findings support the idea that the prevention of calcium homeostasis alteration and of mitochondria dysfunction by hexarelin and JMV2894 could contribute to preserve *in vivo* muscle function.

The molecular mechanisms activated by hexarelin and JMV2894 have not been fully characterized. It is likely that, similarly to ghrelin^6^, they may activate multiple pathways and their effects could occur also in the absence of the GHS-R-1a receptor. The positive effect of hexarelin and JMV2894 at the level of the mitochondrial biogenesis master regulator, PGC-1α, suggests that mitochondria play a pivotal role in these mechanisms. Hexarelin and JMV2894 might stimulate growth hormone (GH) release and this may activate PGC-1α through the IGF1-AKT-mTOR-YY1 pathway. Accordingly, IGF-1 has been proposed as countermeasure against muscle impairment in disuse^61^. PGC-1α may then activate mitochondrial biogenesis, mitochondrial antioxidants and inhibit FoxO3a activity.

The increase of mitochondrial biogenesis due to GHS administration could be a successful compensatory strategy in preventing muscle cachexia. This suggestion is in agreement with a number of reports clearly indicating that the increase of mitochondrial biogenesis can improve respiratory defects in several diseases associated with mitochondrial dysfunctions. Recently we showed that incomplete penetrance in Leber’s hereditary optic neuropathy (LHON) is associated with increased mtDNA content and increased mitochondrial biogenesis^62,63^. Moreover, activation of mitochondrial biogenesis by means of pharmaceutical or natural compounds has been shown to lead to the improvement of respiratory chain defects in murine models of mitochondrial diseases^64,65^.

It should be considered that mitochondrial homeostasis is a complex process in which two opposing pathways, mitochondrial biogenesis and mitophagy tune up the quantity and quality of mitochondria, allowing cells to adapt their mitochondrial content in response to cellular metabolic state, stress and other environmental and cellular signals. An imbalance between the two processes results in functional deterioration of biological systems and promotes cell death.

The analysis of biogenetic and mitophagy markers in our experimental system suggests that cisplatin treatment causes an imbalance between the two pathways leading to an increase of some autophagy markers, defects in lysosome clearance and reduction of mitochondrial number. This imbalance may cause cell death as indicated by the reduction of muscle weight and fiber size. The treatment with the two GHS prevents those changes and appear to restore the balances between the two events, as we observe an increase of mitochondrial biogenesis and mitochondrial mass and a decrease of parameters linked to mitophagy. It is well known that biogenesis and mitophagy are controlled by complex and often interlinked pathways; a detailed study of the factors involved in these processes should provide interesting information on the molecular mechanisms underlying the changes of mitochondrial homeostasis in cachexia and in the presence of GHS. This will offer new hopes for more effective therapeutic strategies for the treatment of cachexia.

In conclusion, here we demonstrate that GHS administration can prevent mitochondrial deficiency in cisplatin-induced cachectic muscle by stimulating the mitochondrial biogenesis and the cellular antioxidant defenses, by ensuring the maintenance of the mitochondrial fission and fusion balance and by preventing accumulation of oxidized protein.

## Materials and Methods

### Animals care and experimental protocol

Animal care and all experimental protocols involving animals were in accordance with the European Directive 2010/63/EU and were approved by the Italian Ministry of Health and by the Committee on Animal Experimentation of the University of Milano-Bicocca. Adult male rats were purchased from Charles River Laboratories (Italy). Rats were housed in a temperature-, humidity-, and light-controlled room. Rats were randomized to receive vehicle (saline, CTR), cisplatin (CIS), cisplatin + hexarelin (HEX), cisplatin + JMV2894 (JMV) using an experimental protocol previously described^18^. Each group consisted of 4 rats. Cisplatin (cis-platinum II-diammine dichloride) was purchased from Sigma-Aldrich (St. Louis, MO, USA). Hexarelin and JMV2894 were synthesized by us as previously described^9,10^. Hexarelin, JMV2894 and cisplatin were freshly dissolved in physiological saline immediately before administration. Cisplatin (1 mg/kg, i.p.) was administered on day 1 through 3 at 9 AM; hexarelin (160 µg/kg) and JMV2894 (320 µg/kg) were injected i.p. twice daily at 8.30 AM (30 min before cisplatin) and 5 PM from day 1 through 5. On day 5, 2 h after hexarelin or JMV2894 injection, rats were sacrificed. This regimen was selected based on previous studies showing: i) cisplatin-induced weight loss without overt-nephrotoxicity^16,18^; ii) a partial prevention of cisplatin-induced weight loss mediated by hexarelin or JMV2894 beginning from day 5 during 12-day GHS treatments duration^17^. Body weight and food intake were assessed daily by weighing the food and the animals before the AM injection. Body weight changes were expressed as change from baseline, and food intake was expressed in g/day. Animals were anesthetized before being sacrificed and the TA muscle was immediately removed, weighed, snap-frozen in isopentane cooled by liquid nitrogen and stored in liquid nitrogen until further use.

### Determination of mtDNA content

Total DNA was extracted from 30–50 mg of each frozen sample of TA skeletal muscle obtained from the four groups of rats with the Wizard Genomic DNA Purification kit (Promega Corporation, Woods Hollow Road Madison, WI, USA) according to the procedures described in the Supplementary Materials.

MtDNA content was determined by quantitative real time PCR (qRT-PCR), via SYBR Green chemistry on a QuantStudio™ 7 Flex Real-Time PCR System (Applied Biosystems, Foster City, CA, USA), amplifying mitochondrial D-loop and β-actin (ACTB) genes. Primer sequences and PCR conditions are described in the Supplementary Materials. The relative quantification of mtDNA was performed according to the Pfaffl mathematical model^66^.

### Isolation of total RNA, reverse transcription and real-time RT-PCR

TA muscles were snap frozen in liquid nitrogen soon after removal and stored at −80 °C until use. For each muscle sample, total RNA was isolated with TRIzol (Life Technologies C.A. 10296028) and quantified by using a spectrophotometer (ND- 1000 Nano-Drop, Thermo Scientific).

To perform reverse transcription, for each sample, 400 ng of total RNA was added to 1 μl dNTP mix 10 mM each, (Roche N.C. 11277049001), 1 μl Random Hexamers 50 μM (Life Technologies C.N. n808–0127) and incubated at 65 °C for 5 min. Afterward, 4 μl 5X First Standard Buffer (Life Technologies C.N. Y02321), 2 μl 0,1 M DTT (Life Technologies C.N. Y00147) and 1 μl Recombinant RNasin Ribonuclease Inhibitor 40 U/μl (Promega C.N. N2511) were added and incubated at 42 °C for 2 min. To each solution, 1 μl Super Script II Reverse Transcriptase 200 U/μl (Life Technologies C.N. 18064–014) was added and incubated at 25 °C for 10 min, at 42 °C for 50 min and at 70 °C for 15 min. Real-time PCR was performed in triplicate using the Applied Biosystems Real-time PCR 7500 Fast system, MicroAmp Fast Optical 96-Well Reaction Plate 0.1 mL (Life Technologies C. N. 4346906) and MicroAmp Optical Adhesive Film (Life Technologies C.N. 4311971). Each reaction was carried in duplicate on a single plex reaction. The setup of reactions consisted 8 ng of cDNA, 0,5 μl of TaqMan Gene Expression Assays, (Life Technologies), 5 μl of TaqMan Universal PCR master mix No AmpErase UNG (2°X) (Life Technologies C.N. 4324018) and Nuclease-Free Water not DEPC-Treated (Life Technologies C. N. AM9930) for a final volume of 10 μl. Under the working RT-TaqMan-PCR conditions: step 1: 95 °C for 20 s; step 2: 95 °C for 3 s; and step 3: 60 °C for 30 s; steps 2 and 3 were repeated 40 times. The results were compared with relative standard curve obtained by five points of 1:4 serial dilutions.

TaqMan Hydrolysis primer and probe gene expression assays were ordered by Life Technologies. To analyze the expression myosin heavy chain isoform 1 (MHC1) encoded by the gene myosin, heavy chain 7 (*Myh7*) we used assay IDs Rn01488777_g1. To analyze the expression myosin heavy chain isoform 2a (MHC2a) encoded by the gene myosin, heavy chain 2, (*Myh2*) we used assay IDs Rn01470656_m1. The mRNA expression of the genes was normalized with *Actinb* (IDs: Rn00667869_m1). The methods of gene expression analysis are the same as those previously used^67^. The RT-PCR experiments were performed in agreement with the MIQE guidelines for qPCR^68^.

### Citrate synthase activity

Total proteins were extracted from ∼50 mg of TA skeletal muscle samples and citrate synthase activity (μmol × min^−1^ × g tissue^−1^) was measured as previously described^69^. Briefly, 20 µg of total proteins were incubated at 37 °C in 1 ml of 0.31 mM acetyl-CoA, 100 mM Tris buffer (pH 8.1), 0.25% Triton X-100, 0.1 mM DTNB and 0.5 mM oxaloacetate. Citrate sinthase activity was determined by measuring the rate of production of thionitrobenzoic acid (TNB) at 412 nm.

### Immunoblot and antibodies

Total proteins were extracted from TA samples obtained from the four groups of rats. Approximately 100 mg of each frozen sample were grounded in liquid nitrogen, followed by resuspension in 220 mM mannitol, 70 mM sucrose, 20 mM Tris–HCl pH 7.4, 1 mM EDTA, 5 mM EGTA, 5 mM MgCl_2_. The suspension was homogenized and centrifuged at 12,000 × g for 4 min, collecting the supernatant. Protein concentration was determined using the Bradford colorimetric method (Bio-Rad Laboratories Inc., Hercules, CA, USA) according to the supplier’s instructions. Immunoblot experiments with antibodies for PGC-1α, NRF-1, TFAM, ND1, Porin, AKT, Phospho-AKT (Ser473), FoxO3a, Phospho-FoxO3a (Ser318/321), PRX III, MnSOD, Drp1, MFN2, Phospho-Drp1 (Ser637), PRX-SO_3_ (PRX-SO_2_H, PRX-SO_3_H), Atrogin-1(Atg1), Beclin1, LC3A, p62 and β-actin were performed as described in Supplementary Materials. Blots were visualized using the ECL Plus Western Blotting Detection Reagents (GE Healthcare, Buckinghamshire, UK) and were acquired by ChemiDoc™ MP Imaging System (Version 5.1) in a single-channel protocol that enabled us to acquire a single image from the blot, in a signal accumulation mode for chemiluminescence. Blot’s images were analyzed with Image Lab™ Software through ‘Lane and Bands’ method.

The evaluation of the relative amount of each protein was performed relating the densitometric value of optical density (OD) units of each protein band to the OD units of the respective β-actin band and normalizing with respect to the samples control group. The phosphorylation level of AKT FoxO3a and Drp1 was determined relating the densitometric value of optical density (OD) units of each phosphorylated protein band to the OD units of the band of the total protein and normalizing with respect to the samples control group.

### Statistical analysis

Differences among experimental groups were determined by one-way analysis of variance (ANOVA) followed by Tukey’s Honestly Significant Difference (HSD) *post hoc* test using the program Statistical Package for the Social Sciences (SPSS) Base 11.5 software (SPSS Inc.,Chicago, IL). Statistical significances are considered as follows: *indicating p ≤ 0.05 and **indicating *p* ≤ 0.01.

## Electronic supplementary material

Supplementary Information

## References

[1] Tisdale MJ (2009). Mechanisms of cancer cachexia. Physiol. Rev..

[2] Fearon K (2011). Definition and classification of cancer cachexia: an international consensus. Lancet Oncol..

[3] Carson JA, Hardee JP, VanderVeen BN (2016). The emerging role of skeletal muscle oxidative metabolism as a biological target and cellular regulator of cancer-induced muscle wasting. Semin. Cell Dev. Biol..

[4] Mantovani G, Madeddu C (2010). Cancer cachexia: medical management. Support Care Cancer..

[5] Anderson LJ, Albrecht ED, Garcia JM (2017). Update on management of cancer-related cachexia. Curr. Oncol. Rep..

[6] Chen J (2015). Ghrelin prevents tumour- and cisplatin-induced muscle wasting: characterization of multiple mechanisms involved. J. Cachexia Sarcopenia Muscle..

[7] Nagaya N (2001). Chronic administration of ghrelin improves left ventricular dysfunction and attenuates development of cardiac cachexia in rats with heart failure. Circulation..

[8] De Vriese C (2004). Ghrelin degradation by serum and tissue homogenates: identification of the cleavage sites. Endocrinology..

[9] Moulin A (2007). Toward potent ghrelin receptor ligands based on trisubstituted 1,2,4-triazole structure. 2. Synthesis and pharmacological *in vitro* and *in vivo* evaluations. J. Med. Chem..

[10] Demange L (2007). Synthesis and pharmacological *in vitro* and *in vivo* evaluations of novel triazole derivatives as ligands of the ghrelin receptor. J. Med. Chem..

[11] Ali S, Chen JA, Garcia JM (2013). Clinical development of ghrelin axis-derived molecules for cancer cachexia treatment. Curr. Opin. Support. Palliat. Care..

[12] Temel JS (2016). Anamorelin in patients with non-small-cell lung cancer and cachexia (ROMANA 1 and ROMANA 2): results from two randomised, double-blind, phase 3 trials. Lancet Oncol..

[13] Barreto R (2016). Cancer and chemotherapy contribute to muscle loss by activating common signaling pathways. Front. Physiol..

[14] Penna F, Busquets S, Argilés JM (2016). Experimental cancer cachexia: Evolving strategies for getting closer to the human scenario. Semin. Cell Dev. Biol..

[15] Garcia JM (2013). Inhibition of cisplatin-induced lipid catabolism and weight loss by ghrelin in male mice. Endocrinology..

[16] Sakai H (2014). Mechanisms of cisplatin-induced muscle atrophy. Toxicol. Appl. Pharmacol..

[17] Bresciani, E. *et al*. JMV2894, a novel growth hormone secretagogue, accelerates body mass recovery in an experimental model of cachexia. *Endocrin*. 10.1007/s12020-016-1184-2 (2016). 10.1007/s12020-016-1184-2 27896546

[18] Conte E (2017). Growth hormone secretagogues prevent dysregulation of skeletal muscle calcium homeostasis in a rat model of cisplatin-induced cachexia. J. Cachexia Sarcopenia Muscle..

[19] Pesce V (2001). Age-related mitochondrial genotypic and phenotypic alterations in human skeletal muscle. Free Radic. Biol. Med..

[20] Picca A (2014). A comparison among the tissue-specific effects of aging and calorie restriction on TFAM amount and TFAM-binding activity to mtDNA in rat. Biochim. Biophys. Acta..

[21] Romanello V, Sandri M (2016). Mitochondrial quality control and muscle mass maintenance. Front. Physiol..

[22] Carter HN, Chen CC, Hood DA (2015). Mitochondria, muscle health, and exercise with advancing age. Physiology (Bethesda)..

[23] Julienne CM (2012). Cancer cachexia is associated with a decrease in skeletal muscle mitochondrial oxidative capacities without alteration of ATP production efficiency. J. Cachexia Sarcopenia Muscle..

[24] Fontes-Oliveira CC (2013). Mitochondrial and sarcoplasmic reticulum abnormalities in cancer cachexia: altered energetic efficiency?. Biochim. Biophys. Acta..

[25] Isaac ST, Tan TC, Polly P (2016). Endoplasmic reticulum stress, calcium dysregulation and altered protein translation: intersection of processes that contribute to cancer cachexia induced skeletal muscle wasting. Curr. Drug Targets..

[26] Barreto R (2016). Chemotherapy-related cachexia is associated with mitochondrial depletion and the activation of ERK1/2 and p38 MAPKs. Oncotarget..

[27] Attaix D, Pichard C, Baracos VE (2015). Muscle wasting: is mitochondrial dysfunction a key target?. Curr. Opin. Clin. Nutr. Metab. Care..

[28] Kelly DP, Scarpulla RC (2004). Transcriptional regulatory circuits controlling mitochondrial biogenesis and function. Genes Dev..

[29] Picca A, Lezza AM (2015). Regulation of mitochondrial biogenesis through TFAM-mitochondrial DNA interactions: useful insights from aging and calorie restriction studies. Mitochondrion..

[30] Malarkey CS (2016). The sea urchin mitochondrial transcription factor A binds and bends DNA efficiently despite its unusually short C-terminal tail. Mitochondrion..

[31] Gustafsson CM, Falkenberg M, Larsson NG (2016). Maintenance and expression of mammalian mitochondrial DNA. Annu. Rev. Biochem..

[32] Cormio A (2012). Mitochondrial DNA content and mass increase in progression from normal to hyperplastic to cancer endometrium. BMC Res. Notes..

[33] Shadel GS, Horvath TL (2015). Mitochondrial ROS signaling in organismal homeostasis. Cell..

[34] Hayashi G, Cortopassi G (2015). Oxidative stress in inherited mitochondrial diseases. Free Radic. Biol. Med..

[35] Powers SK, Wiggs MP, Duarte JA, Zergeroglu AM, Demirel HA (2012). Mitochondrial signaling contributes to disuse muscle atrophy. Am. J. Physiol. Endocrinol. Metab..

[36] Sorensen JC (2016). Mitochondria: inadvertent targets in chemotherapy-induced skeletal muscle toxicity and wasting?. Cancer Chemother. Pharmacol..

[37] Musicco C (2009). Accumulation of overoxidized Peroxiredoxin III in aged rat liver mitochondria. Biochim. Biophys. Acta..

[38] Zhong N, Xu J (2008). Synergistic activation of the human MnSOD promoter by DJ-1 and PGC-1α: regulation by SUMOylation and oxidation. Hum. Mol. Genet..

[39] Valle I, Alvarez-Barrientos A, Arza E, Lamas S, Monsalve M (2005). PGC-1alpha regulates the mitochondrial antioxidant defense system in vascular endothelial cells. Cardiovasc. Res..

[40] Shum AMY (2012). Disruption of MEF2C signaling and loss of sarcomeric and mitochondrial integrity in cancer-induced skeletal muscle wasting. Aging (Albany NY)..

[41] Nan J (2017). Molecular regulation of mitochondrial dynamics in cardiac disease. Biochim. Biophys. Acta..

[42] Sandri M (2004). Foxo transcription factors induce the atrophy-related ubiquitin ligase atrogin-1 and cause skeletal muscle atrophy. Cell..

[43] Mele A (2014). Dual response of the KATP channels to staurosporine: a novel role of SUR2B, SUR1 and Kir6.2 subunits in the regulation of the atrophy in different skeletal muscle phenotypes. Biochem. Pharmacol..

[44] Funderburk SF, Wang QJ, Yue Z (2010). The Beclin 1 -VPS34 complex- at the crossroads of autophagy and beyond. Trends Cell Biol..

[45] Schuler M (2006). PGC1α expression is controlled in skeletal muscles by PPARβ, whose ablation results in fiber-type switching, obesity, and type 2 diabetes. Cell Metabolism..

[46] Sharma RP, Edwards IR (1983). Cis-platinum: subcellular distribution and binding to cytosolic ligands. Biochem. Pharmacol..

[47] Garrido N (2008). Cisplatin-mediated impairment of mitochondrial DNA metabolism inversely correlates with glutathione levels. Biochem. J..

[48] White JP (2012). IL-6 regulation on skeletal muscle mitochondrial remodeling during cancer cachexia in the ApcMin/+mouse. Skelet. Muscle..

[49] Pesce V (2010). Acetyl-L-carnitine supplementation to old rats partially reverts the age-related mitochondrial decay of soleus muscle by activating peroxisome proliferator-activated receptor gamma coactivator-1alpha-dependent mitochondrial biogenesis. Rejuvenation Res..

[50] Wagner E (2002). A method for detection of overoxidation of cysteines: peroxiredoxins are oxidized *in vivo* at the active-site cysteine during oxidative stress. Biochem. J..

[51] Rossi AE, Boncompagni S, Dirksen RT (2009). Sarcoplasmic reticulum-mitochondrial symbiosis: bidirectional signaling in skeletal muscle. Exerc. Sport Sci. Rev..

[52] Pietrangelo L (2015). Age-dependent uncoupling of mitochondria from Ca^2+^ release units in skeletal muscle. Oncotarget..

[53] Klionsky DJ (2016). Guidelines for the use and interpretation of assays for monitoring autophagy (3rd edition. Autophagy..

[54] Penna F, Baccino FM, Costelli P (2014). Coming back: autophagy in cachexia. Curr. Opin. Clin. Nutr. Metab. Care..

[55] Aversa Z (2016). Autophagy is induced in the skeletal muscle of cachectic cancer patients. Sci. Rep..

[56] Wenz T, Rossi SG, Rotundo RL, Spiegelman BM, Moraes CT (2009). Increased muscle PGC-1α expression protects from sarcopenia and metabolic disease during aging. Proc. Natl. Acad. Sci. USA..

[57] Sandri M (2013). Protein breakdown in muscle wasting: role of autophagy-lysosome and ubiquitine proteasome. Int. J. Biochem. Cell Biol..

[58] Sakuma K (2016). p62/SQSTM1 but not LC3 is accumulated in sarcopenic muscle of mice. J. Cachexia Sarcopenia Muscle..

[59] Carnio S (2014). Autophagy impairment in muscle induces neuromuscular junction degeneration and precocious aging. Cell Reports.

[60] Liantonio A (2013). Growth hormone secretagogues exert differential effects on skeletal muscle calcium homeostasis in male rats depending on the peptidyl/nonpeptidyl structure. Endocrinol..

[61] Tamaki M (2015). Improvement of physical decline through combined effects of muscle enhancement and mitochondrial activation by a gastric hormone ghrelin in male 5/6Nx CKD model mice. Endocrinology..

[62] Giordano C (2014). Efficient mitochondrial biogenesis drives incomplete penetrance in Leber’s hereditary optic neuropathy. Brain..

[63] Giordano L (2015). Cigarette toxicity triggers Leber’s hereditary optic neuropathy by affecting mtDNA copy number, oxidative phosphorylation and ROS detoxification pathways. Cell Death Dis..

[64] Viscomi C (2011). *In vivo* correction of COX deficiency by activation of the AMPK/PGC-1α axis. Cell. Metab..

[65] Cerutti R (2014). NAD(+)-dependent activation of Sirt1 corrects the phenotype in a mouse model of mitochondrial disease. Cell Metab..

[66] Pfaffl MW (2001). A new mathematical model for relative quantification in real-time RT-PCR. Nucleic Acids Res..

[67] Pierno S (2013). Paracrine effects of IGF-1 overexpression on the functional decline due to skeletal muscle disuse: molecular and functional evaluation in hindlimb unloaded MLC/mIgf-1 transgenic mice. PLoS One..

[68] Bustin SA (2009). The MIQE guidelines: minimum information for publication of quantitative real-time PCR experiments. Clin. Chem..

[69] Srere PA (1969). Cytrate synthase. Methods Enzymol..

