# Supplementary material for: Growth hormone secretagogues hexarelin and JMV2894 protect skeletal muscle from mitochondrial damages in a rat model of cisplatin-induced cachexia
Source: Sci Rep. 2017 Oct 12;7:13017. doi: 10.1038/s41598-017-13504-y (PMC5638899; doi:10.1038/s41598-017-13504-y)
Supplement: Supplementary file 1 — Supplementary Information [file 41598_2017_13504_MOESM1_ESM.pdf]

SUPPLEMENTARY INFORMATION

Growth hormone secretagogues hexarelin and JMV2894 protect skeletal muscle from mitochondrial damages in a rat model of cisplatin-induced cachexia

Giuseppe Sirago[1], Elena Conte[2], Flavio Fracasso[1], Antonella Cormio[1], Jean-Alain Fehrentz[3], Jean Martinez[3], Clara Musicco[4], Giulia Maria Camerino[2], Adriano Fonzino[2], Laura Rizzi[5], Antonio Torsello[5], Angela Maria Serena Lezza[1], Antonella Liantonio[2], Palmiro Cantatore[1] & Vito Pesce[1,*]

[1] Department of Biosciences, Biotechnologies and Biopharmaceutics, University of Bari "A. Moro", Bari, Italy.
[2] Department of Pharmacy-Drug Sciences, University of Bari "A. Moro", Bari, Italy.
[3] Max Mousseron Institute of Biomolecules UMR5247, CNRS, University of Montpellier, ENSCM, Montpellier, France.
[4] IBBE Institute of Biomembranes and Bioenergetics CNR- National Research Council of Italy, Bari, Italy.
[5] School of Medicine and Surgery, University of Milano-Bicocca, Monza, Italy.

*****Corresponding author:

Vito Pesce, Department of Biosciences, Biotechnologies and Biopharmaceutics, University of Bari "A. Moro", Bari, Italy (e.mail:vito.pesce@uniba.it).

SUPPLEMENTARY MATERIAL

SUPPLEMENTARY MATERIALS AND METHODS

Determination of mtDNA content.

Total DNA was extracted from 30-50 mg of each frozen sample of TA skeletal muscle obtained from the four groups of rats by means of the Wizard Genomic DNA Purification kit (Promega Corporation, Woods Hollow Road Madison, WI, USA). Approximately 30-50 mg of each frozen sample was grounded in liquid nitrogen, suspended in 1 ml of Nuclei Lysis Solution, homogenized and incubated for 30 minutes at 65°C. The RNA in the extract was degraded by incubating the sample at 37°C for 30 minutes with RNase A Solution (3µl) and proteins were precipitated by adding 200 µl of Protein Precipitation Solution. Samples were centrifuged at 13,000g for 2 minutes and the supernatant was precipitated with 600 µl of isopropanol and centrifuged. The obtained pellet was washed with 70% ethanol. After ~~a~~ centrifugation, the pellet was suspended in sterile water. At the end of the extraction, the DNA concentration was determined by reading the absorbance at 260 nm. The extracted DNA was run on a 0.8% agarose gel in TAE 1X to check the quality of extraction. DNA samples were frozen at -20°C until further use.

MtDNA content was determined by means of quantitative Real Time PCR (qRT-PCR), via SYBR Green chemistry on a QuantStudio™ 7 Flex Real-Time PCR System (Applied Biosystems, Foster City, CA, USA), amplifying non-coding displacement (D) loop of mitochondrial DNA (D-loop) and actin beta (ACTB) gene.

The primers were specific, respectively, for the rat mitochondrial D-loop region (D-loop For: 5’-GGTTCTTACTTCAGGGCCATCA-3’; D-loop Rev: 5’-TGATTAGACCCGTTACCATCGA-3’, accession number AY172581) and for the rat nuclear β-actin gene (β-actin For: 5’-CCCAGCCATGTACGTAGCCA-3’; β-actin Rev: 5’-CGTGTCCGGAGTCCATCAC-3’, accession number VO1217.1). The method was validated by primer-limiting experiments and by assessing the similar reaction efficiency of the two amplicons. Amplification specificity was controlled by melting curve analysis and gel electrophoresis. Each sample was analyzed in triplicate in 20 µl of final volume containing: SYBR Select Master Mix 1X (Applied Biosystems, Foster City, CA, USA), 0.2 µM forward and reverse primers, and DNA template (25 ng). After 5 minutes of denaturation at 95°C, amplification proceeded for 40 cycles, each consisting of denaturation at 95°C for 3 s, annealing, and extension at 60°C for 30 s. The quantification of the mtDNA content was performed according to the Pfaffl mathematical model (Pfaffl, 2001).

Immunoblot and antibodies

Total proteins were extracted from TA samples obtained from the four groups of rats. Approximately 100 mg of each frozen sample were grounded in liquid nitrogen and suspended in

500 μl of lysis buffer containing 220 mM Mannitol, 70 mM Sucrose, 20 mM Tris-HCl pH 7.4, 1mM EDTA, 5 mM EGTA, 5 mM $MgCl_2$. The mixture was homogenized and centrifuged at 12,000g for 4 minutes, collecting the supernatant. Protein concentration was determined using the Bradford colorimetric method (Bio-Rad Laboratories Inc., Hercules, CA, USA) according to the supplier's instructions.

Ten micrograms of proteins from each animal of the four experimental groups were separated by a 4-12% Bis-Tris sodium dodecyl sulfate polyacrylamide gel electrophoresis (SDS-PAGE) (Criterion™ XT Precast Gel, Bio-Rad Laboratories Inc., Hercules, CA, USA) and then electroblotted for 3 hours onto polyvinylidene fluoride (PVDF) membranes Hybond-P (GE Healthcare, Buckinghamshire, UK), using Criterion™ Blotter (Bio-Rad Laboratories Inc, Hercules, CA). Each gel contained a lane with Molecular Weight marker (PageRuler™ Prestained Protein Ladder; Thermo Fisher Scientific, Waltham, MA, USA). After protein transfer, the membranes were incubated overnight at 4°C with primary antibodies for PGC-1α, AKT, Phospho-AKT (Ser473), FoxO3a, Phospho-FoxO3a (Ser318/321), MFN2, Atrogin 1, Beclin 1, LC3A, ND1, Porin, Drp1 and Phospho-DRP1 (Ser637) in 5% w/v BSA, 1X TBS, 0.1% Tween® 20. For TFAM, PRX III, MnSOD, oxidized PRXs (Ox PRXs, PRX-SO_3H), β-actin, p62, and NRF-1 the incubation mixture contained 1% w/v powdered milk, 1X PBS, 0.1% Tween® 20. The next day, membranes were incubated for 1 hr with appropriate peroxidase-conjugated secondary antibodies (Santa Cruz Biotechnology-Santa Cruz, CA).

Blots were visualized using the ECL Plus Western Blotting Detection Reagents (GE Healthcare, Buckinghamshire, UK) and were acquired by ChemiDoc™ MP Imaging System (Version 5.1) in a single-channel protocol that enabled us to acquire a single image from the blot, in a signal accumulation mode for chemiluminescence. Blot's images were analyzed with Image Lab™ Software through 'Lane and Bands' method.

Before to perform the protein quantification experiments each antibody was tested separately verifying the presence of a single immunoreactive band of the expected molecular weight for all of them (see Supplementary Figure 1). Having verified the migration of all the immunoreacted bands the filter was cut in various slices after the transfer, having care to include a space of at least 1 cm above and 1 cm below the expected band. In this way the same filter could be simultaneously probed with different antibodies. Since we analyzed a remarkable number of proteins this allowed to save time and material.

The evaluation of the relative amount of each analyzed protein was performed relating the densitometric value of optical density (OD) units of each protein band to the OD units of the respective β-actin band and normalizing with respect to the samples control group. The

phosphorylation level of AKT and FoxO3a was determined relating the densitometric value of optical density (OD) units of each phosphorylated protein band to the OD units of the band of the total protein and normalizing with respect to the samples control group. The data reported in all histograms are the average of at least 4 independent experiments performed on 4 rats for each treatment.

Supplementary Table S1.

Antibodies included in the study

Antibody	Protein M .W.	Company Antibody (ID)	Description	Primary Ab Dilution	Secondary Ab Dilution
PGC-1α	95 KDa	Santa Cruz Biotechnology (sc-5816)	Goat Polyclonal	1:10,000	1:10,000
NRF-1	72 KDa	Santa Cruz Biotechnology (sc-33771)	Rabbit Polyclonal	1:5,000	1:10,000
TFAM	25 KDa	Santa Cruz Biotechnology (sc-19050)	Goat Polyclonal	1:60,000	1:20,000
ND1	32 KDa	Santa Cruz Biotechnology (sc-65237)	Mouse Monoclonal	1:1,000	1:5,000
Porin	32 KDa	Abcam (ab-34726)	Rabbit Polyclonal	1:10,000	1:10,000
PRX-SO_3	28 KDa	Ab Frontier (LF-PA0004)	Rabbit Polyclonal	1:10,000	1:20,000
MnSOD	26 KDa	Assay Designs (SOD-110)	Rabbit Polyclonal	1:50,000	1:20,000
PRX III	26 KDa	Ab Frontier (LF-PA0030)	Rabbit Polyclonal	1:10,000	1:20,000
MFN2	80 KDa	Abnova (H00009927-M03)	Mouse Monoclonal	1:10,000	1:10,000
DRP1	80 KDa	Abnova (H00010059-M01)	Mouse Monoclonal	1:10,000	1:20,000
p-DRP1	80 KDa	Cell Signaling (sc-4867)	Rabbit Polyclonal	1:500	1:10,000
AKT	60 KDa	Cell Signaling (sc-9272)	Rabbit Polyclonal	1:6,000	1:10,000
p-AKT	60 KDa	Cell Signaling (sc-9271)	Rabbit Polyclonal	1:2,500	1:20,000
FoxO3a	97 KDa	Cell Signaling (sc-9467)	Rabbit Polyclonal	1:5,000	1:10,000
p-FoxO3a	97 KDa	Cell Signaling (sc-9465)	Rabbit Polyclonal	1:2,500	1:20,000
Atrogin 1	42 KDa	Santa Cruz Biotechnology (sc-33782)	Rabbit Polyclonal	1:10,000	1:10,000
Beclin 1	55 KDa	Millipore (AB15417)	Rabbit Polyclonal	1:80,000	1:20,000
p62	62 KDa	Sigma-Aldrich (P0066)	Rabbit Monoclonal	1:10,000	1:10,000
LC3A I<br>LC3A II	18 KDa<br>16 KDa	Sigma-Aldrich (L8793)	Rabbit Polyclonal	1:5,000	1:10,000
β-actin	42 KDa	Sigma-Aldrich (A2066)	Rabbit Polyclonal	1:50,000	1:20,000

SUPPLEMENTARY FIGURE LEGENDS

Supplementary Figure 1 (A-Q). Antibody testing.

Supplementary Figure 2 (A-P). Representative whole blots of proteins used in manuscript. The lanes enclosed in the boxes are those that are reported in the figures of the manuscript.

Supplementary figure 1 (A)

PGC-1α

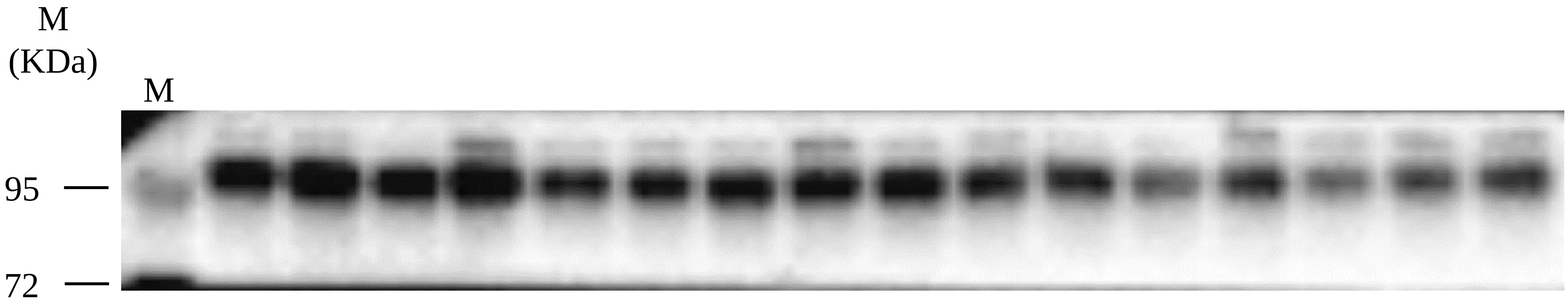

Supplementary Figure S1 (A). PGC-1α protein antibody testing.
M = Marker Page Ruler Prestained Protein Ladder (Thermo Scientific).

Supplementary figure 1 (B)

NRF-1

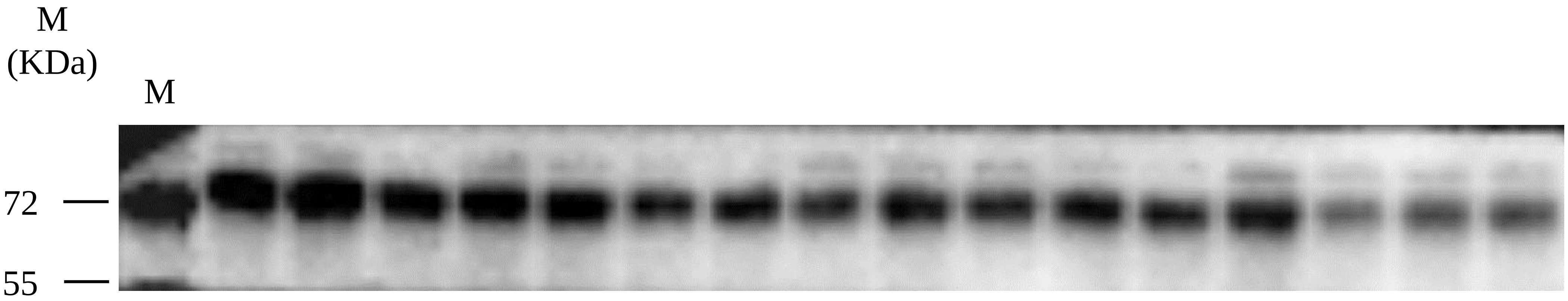

Supplementary Figure S1 (B). NRF-1 protein antibody testing.
M = Marker Page Ruler Prestained Protein Ladder (Thermo Scientific).

Supplementary figure 1 (C)

TFAM

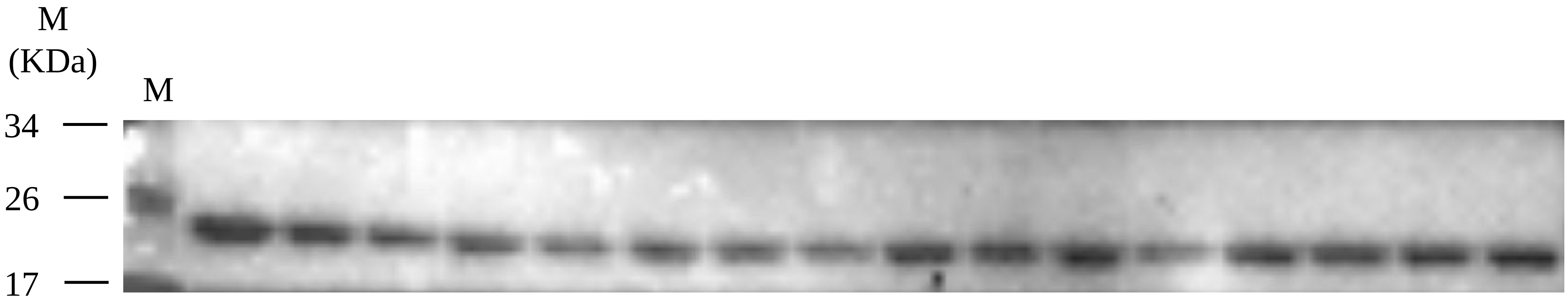

Supplementary Figure S1 (C). TFAMprotein antibody testing.
M = Marker Page Ruler Prestained Protein Ladder (Thermo Scientific).

Supplementary figure 1 (D)

ND1

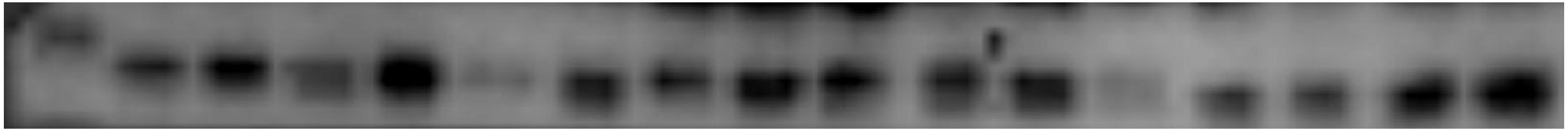

Supplementary Figure S1 (D). ND1 protein antibody testing.
M = Marker Page Ruler Prestained Protein Ladder (Thermo Scientific).

Supplementary figure 1 (E)

Porin

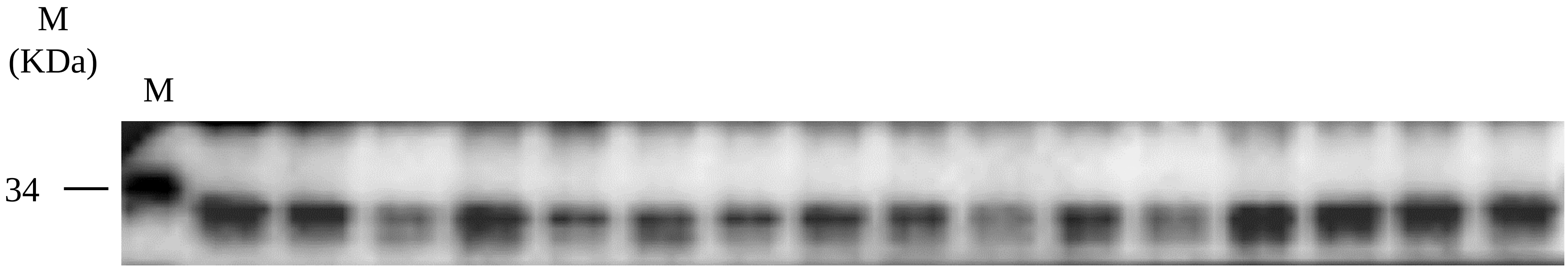

Supplementary Figure S1 (E). Porin protein antibody testing.
M = Marker Page Ruler Prestained Protein Ladder (Thermo Scientific).

Supplementary figure 1 (F)

PRX-SO_3

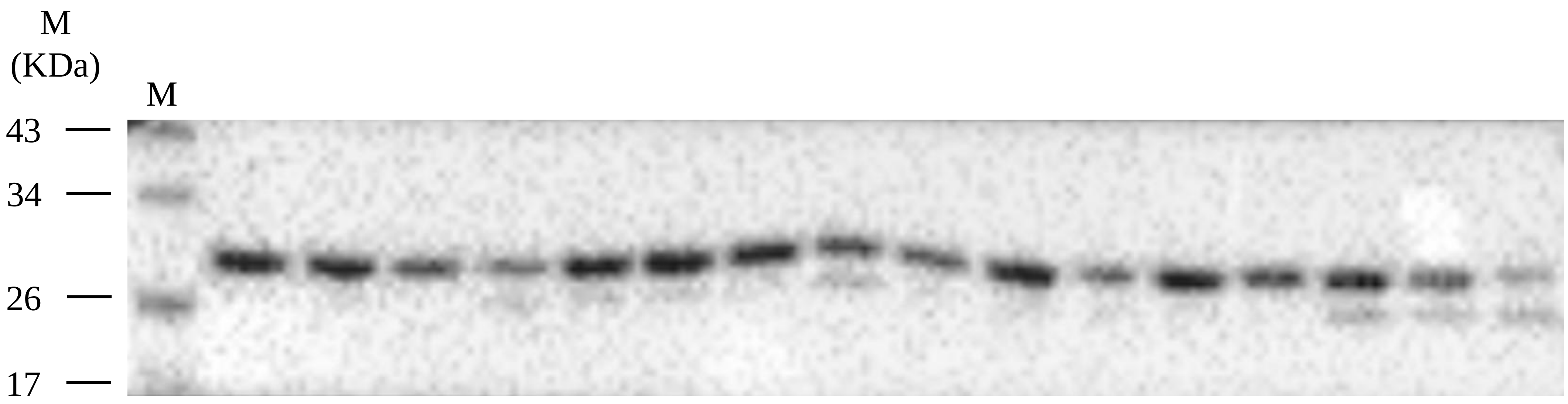

Supplementary Figure S1 (F). PRX-SO_3 protein antibody testing.
M = Marker Page Ruler Prestained Protein Ladder (Thermo Scientific).

Supplementary figure 1 (G)

MnSOD

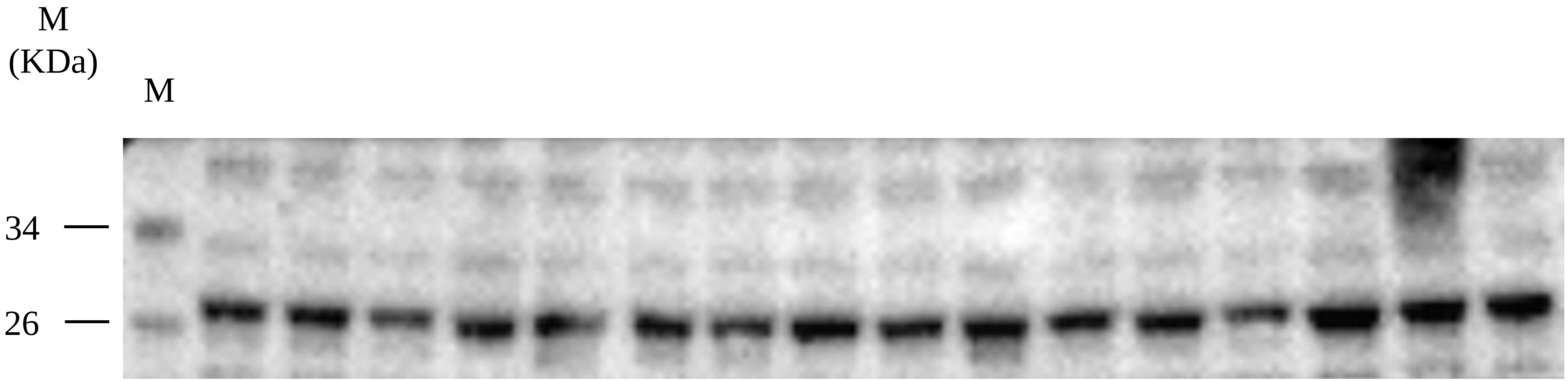

Supplementary Figure S1 (G). MnSOD protein antibody testing.
M = Marker Page Ruler Prestained Protein Ladder (Thermo Scientific).

Supplementary figure 1 (H)

PRX III

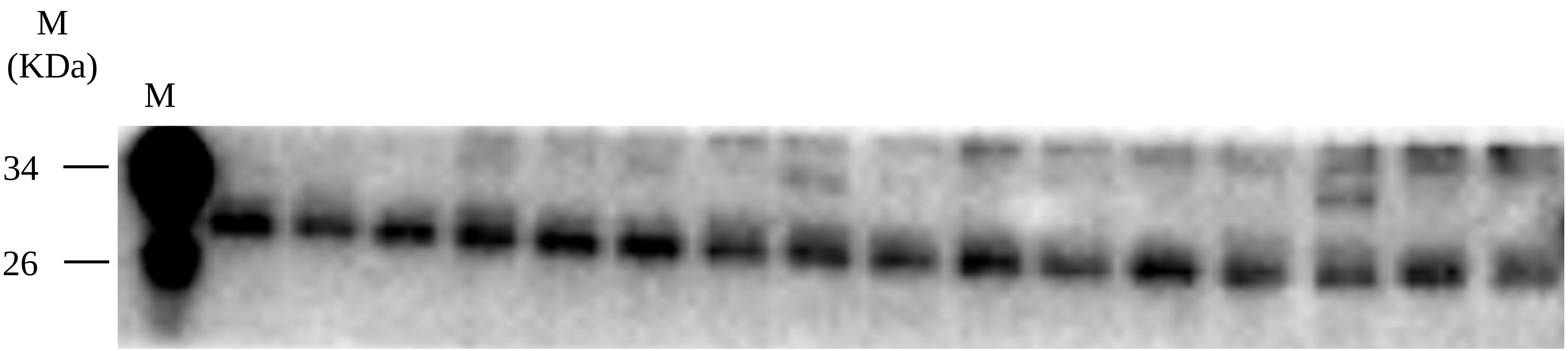

Supplementary Figure S1 (H). PRX III protein antibody testing.
M = Marker Page Ruler Prestained Protein Ladder (Thermo Scientific).

Supplementary figure 1 (I)

MFN2

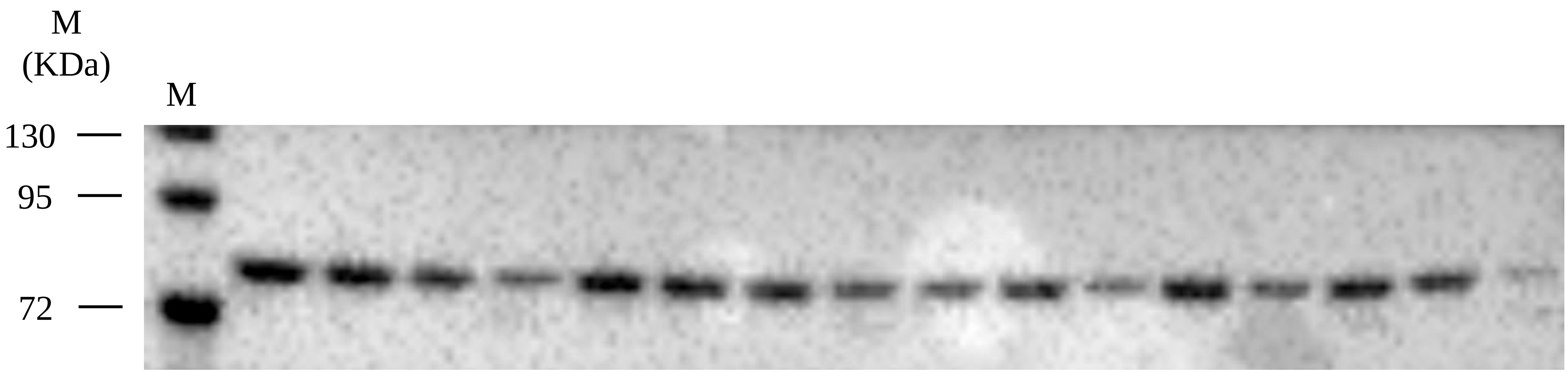

Supplementary Figure S1 (I). MFN2 protein antibody testing.
M = Marker Page Ruler Prestained Protein Ladder (Thermo Scientific).

Supplementary figure 1 (J)

Drp1

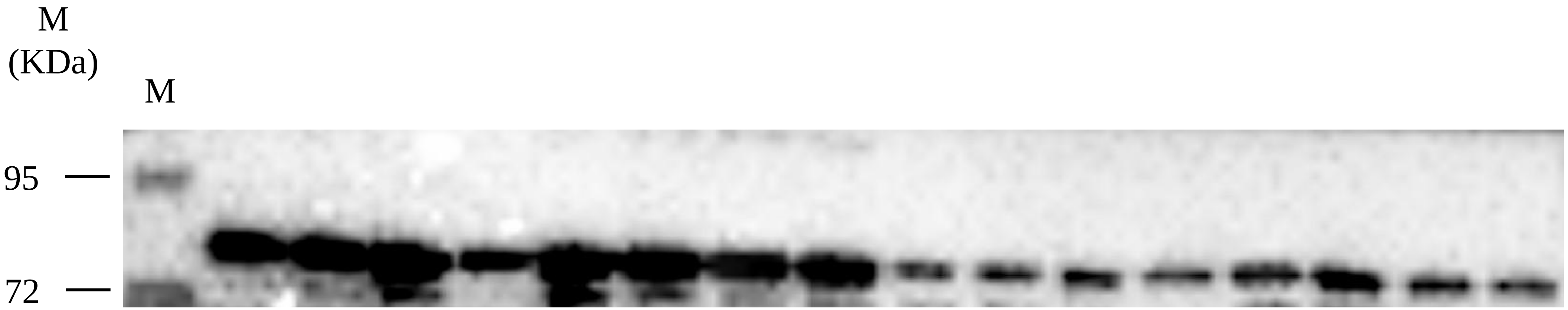

Supplementary Figure S1 (J). Drp1 protein antibody testing.
M = Marker Page Ruler Prestained Protein Ladder (Thermo Scientific).

Supplementary figure 1 (K)

AKT and p-AKT (Ser473)

AKT

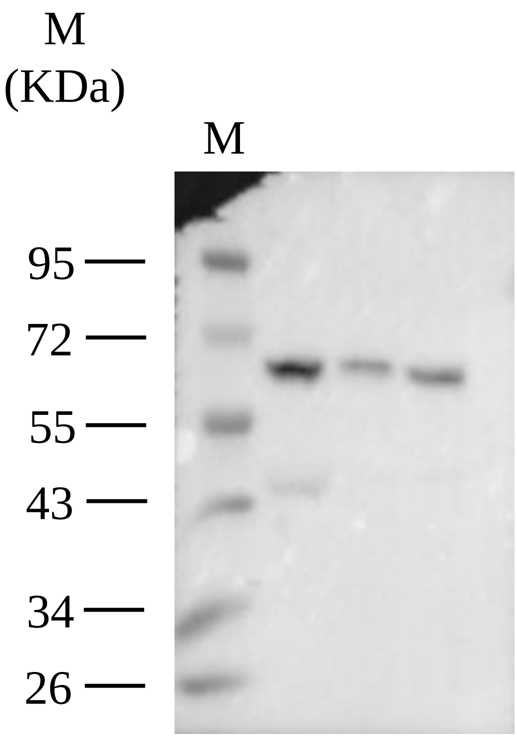

p-AKT (Ser473)

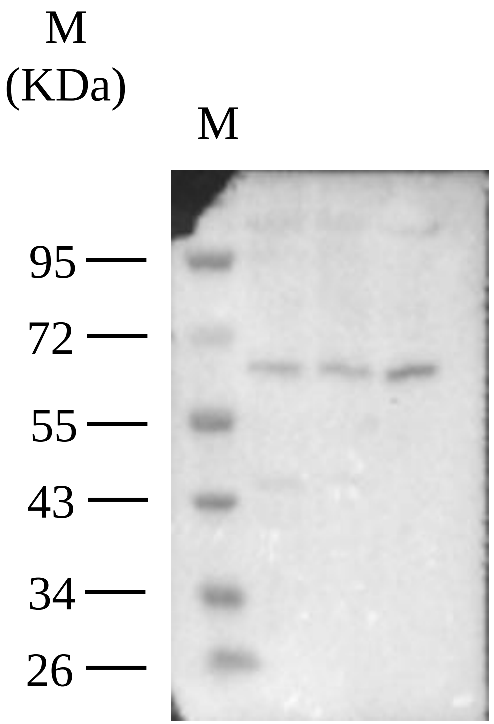

Supplementary Figure S1 (K). AKT and p-AKT (Ser473) proteins antibodies testing.
M = Marker Page Ruler Prestained Protein Ladder (Thermo Scientific).

Supplementary figure 1 (L)

FoxO3a and p-FoxO3a (Ser318/321)

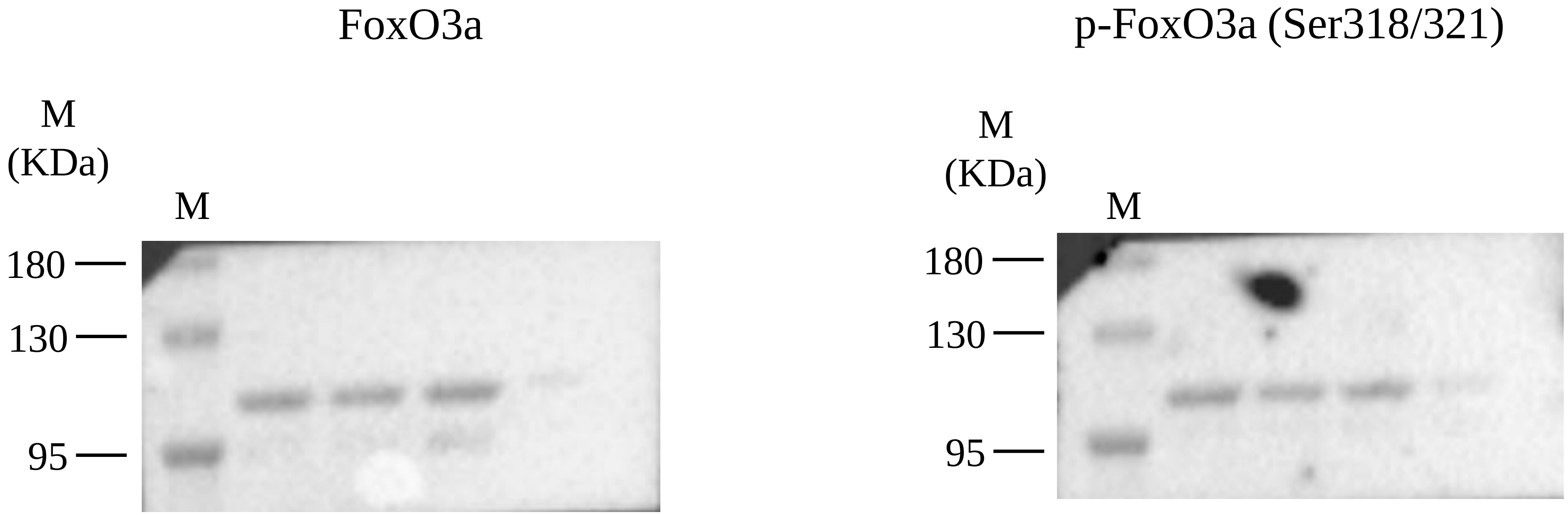

Supplementary Figure S1 (L). FoxO3a and p-FoxO3a (Ser318/321) proteins antibodies testing.
M = Marker Page Ruler Prestained Protein Ladder (Thermo Scientific).

Supplementary figure 1 (M)

Atrogin 1

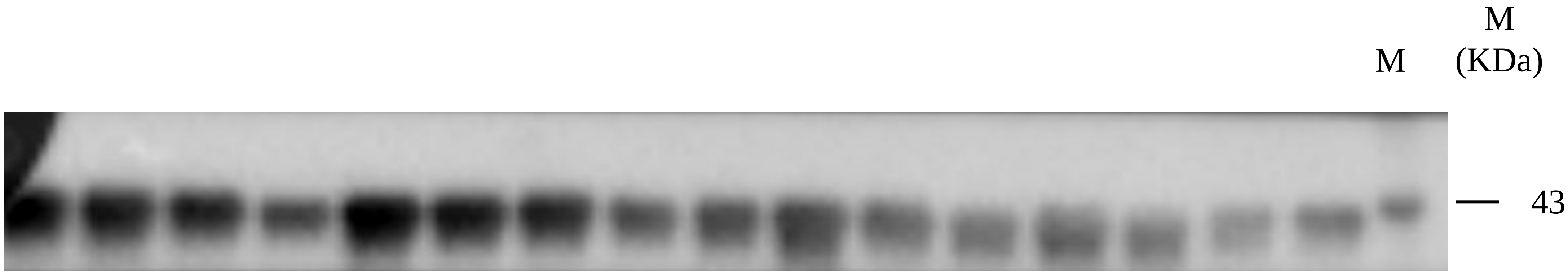

Supplementary Figure S1 (M). Atrogin 1 protein antibody testing.
M = Marker Page Ruler Prestained Protein Ladder (Thermo Scientific).

Supplementary figure 1 (N)

Beclin 1

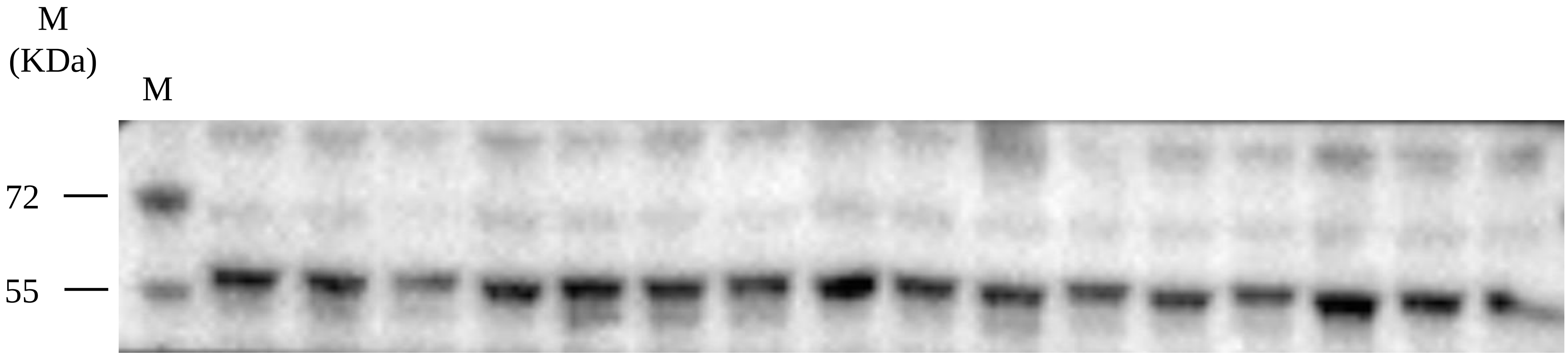

Supplementary Figure S1 (N). Beclin 1 protein antibody testing.
M = Marker Page Ruler Prestained Protein Ladder (Thermo Scientific).

Supplementary figure 1 (O)

LC3 A

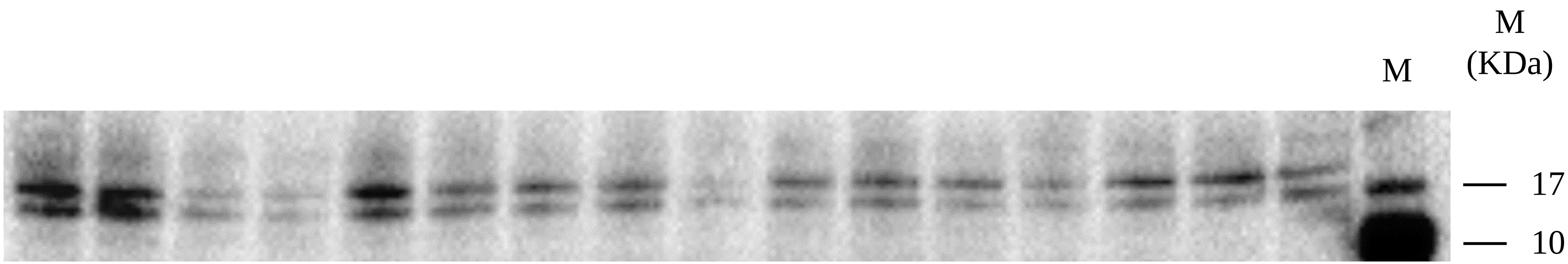

Supplementary Figure S1 (O). LC3A protein antibody testing.
M = Marker Page Ruler Prestained Protein Ladder (Thermo Scientific).

Supplementary figure 1 (P)

p62

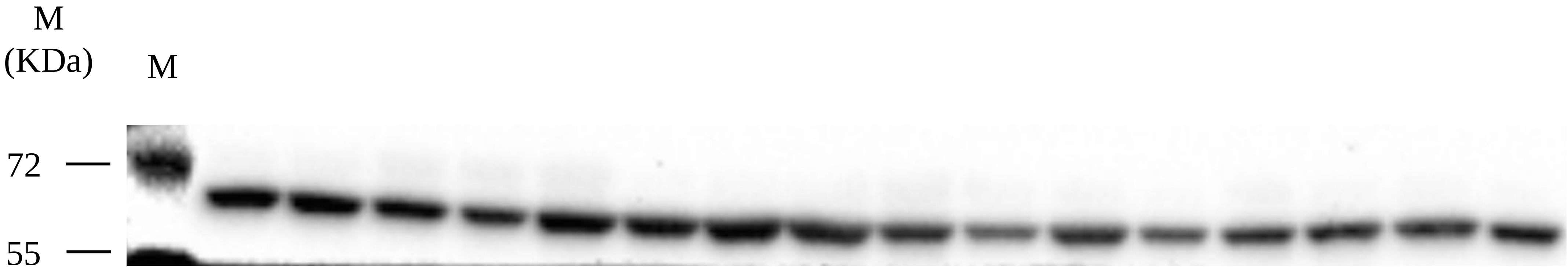

Supplementary Figure S1 (P). p62 protein antibody testing.
M = Marker Page Ruler Prestained Protein Ladder (Thermo Scientific).

Supplementary figure 1 (Q)

β-actin

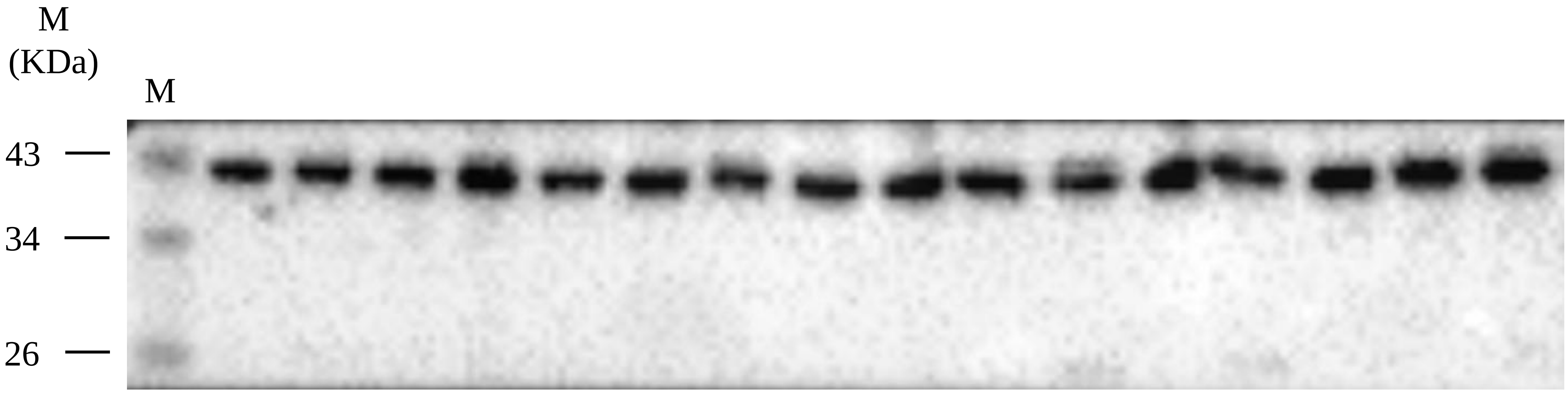

Supplementary Figure S1 (Q). β-actin protein antibody testing.
M = Marker Page Ruler Prestained Protein Ladder (Thermo Scientific).

Supplementary figure 2 (A)

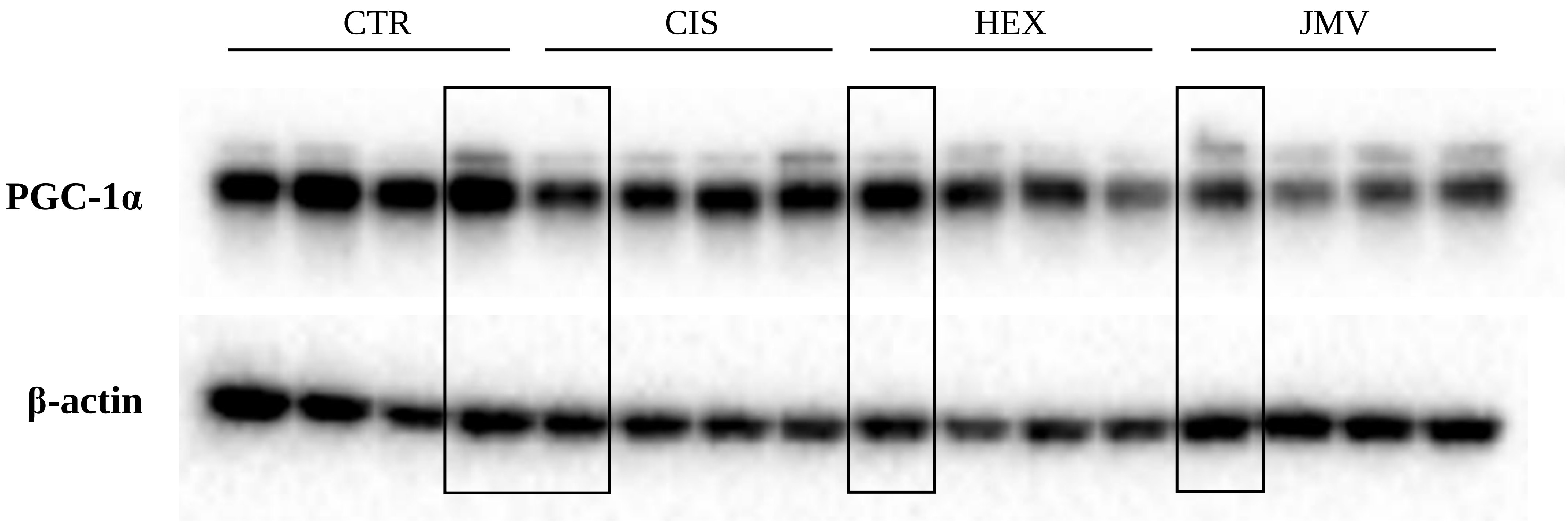

Supplementary Figure S2 (A). Representative western blotting for PGC-1α and β-actin in tibialis anterior rat muscle. CTR, controls; CIS, rats treated with cisplatin; HEX, rats treated with cisplatin and Hexarelin; JMV, rats treated with cisplatin and JMV2894.
The bands enclosed in the boxes are reported in Figure 1(A).

Supplementary figure 2 (B)

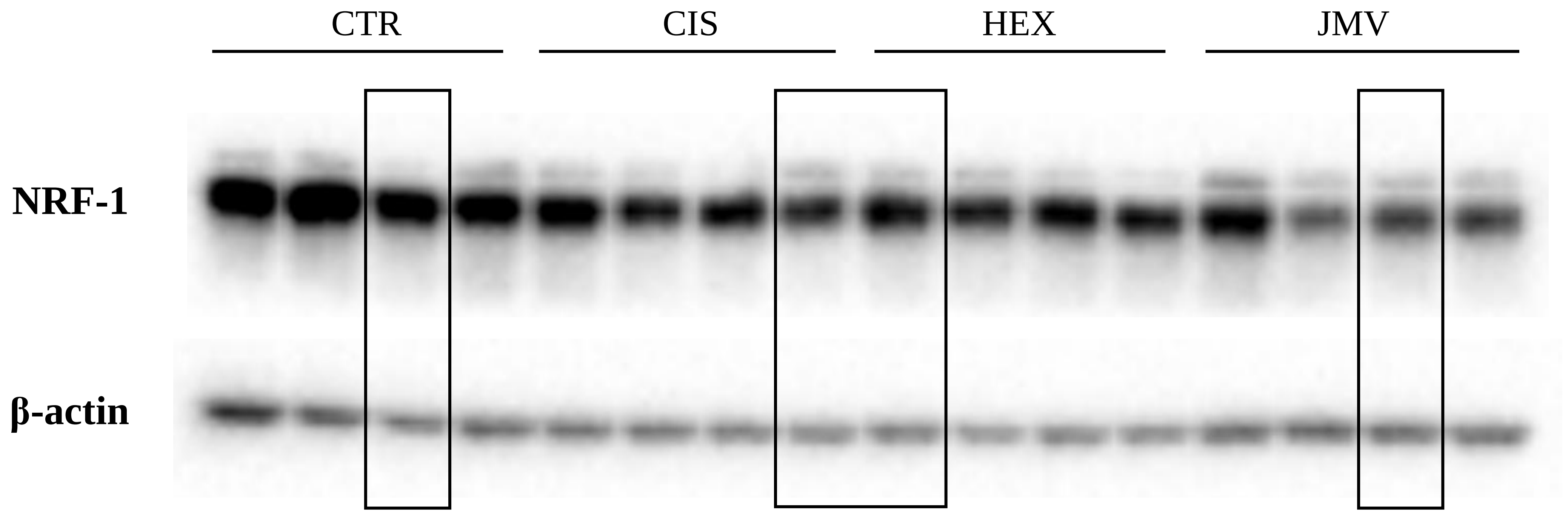

Supplementary Figure S2 (B). Representative western blotting for NRF-1 and β-actin in tibialis anterior rat muscle. CTR, controls; CIS, rats treated with cisplatin; HEX, rats treated with cisplatin and Hexarelin; JMV, rats treated with cisplatin and JMV2894.
The bands enclosed in the boxes are reported in Figure 1(B).

Supplementary figure 2 (C)

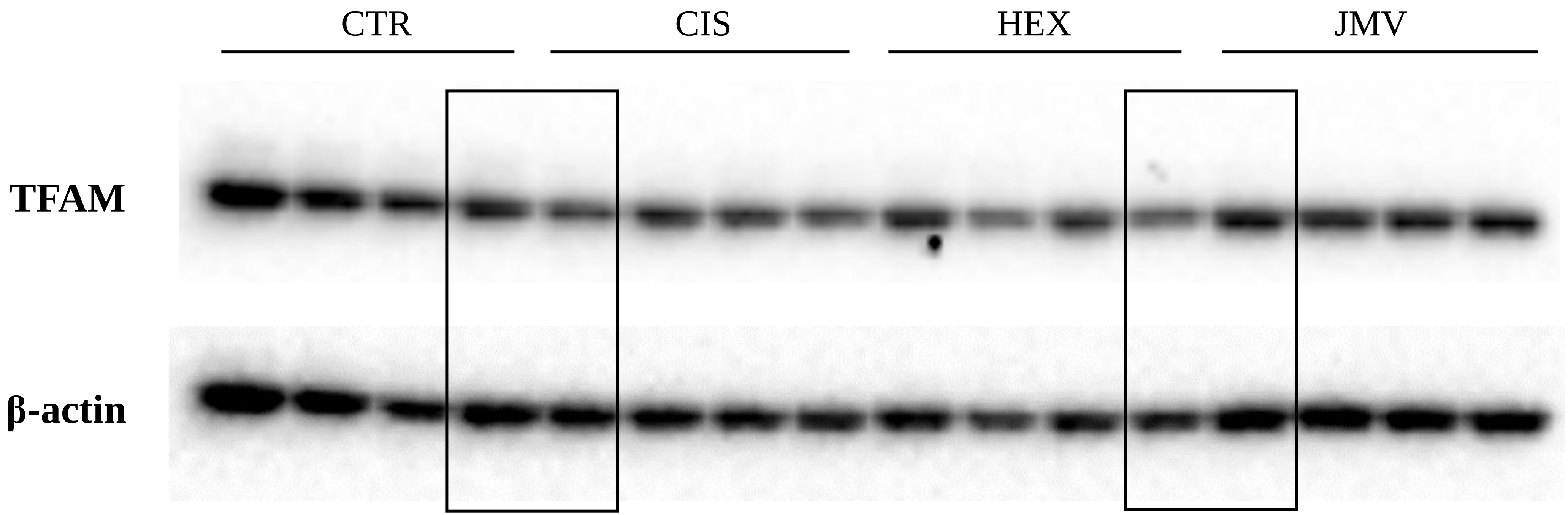

Supplementary Figure S2 (C). Representative western blotting for TFAM and β-actin in tibialis anterior rat muscle. CTR, controls; CIS, rats treated with cisplatin; HEX, rats treated with cisplatin and Hexarelin; JMV, rats treated with cisplatin and JMV2894.
The bands enclosed in the boxes are reported in Figure 1(C).

Supplementary figure 2 (D)

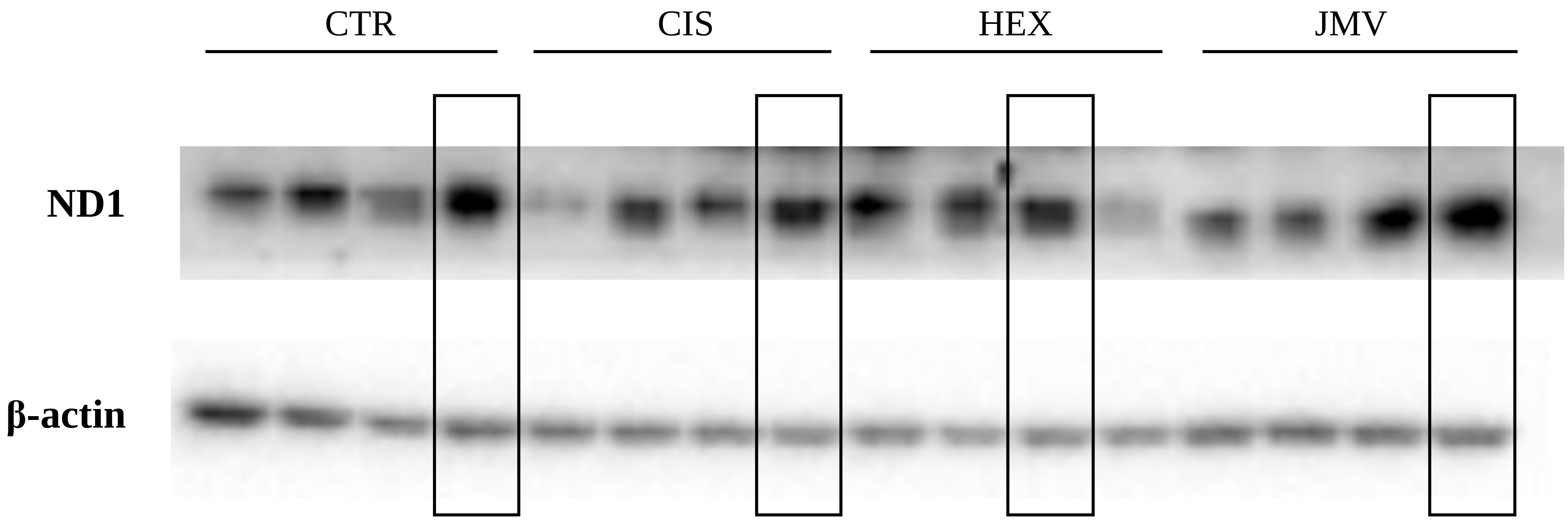

Supplementary Figure S2 (D). Representative western blotting for ND1 and β-actin in tibialis anterior rat muscle. CTR, controls; CIS, rats treated with cisplatin; HEX, rats treated with cisplatin and Hexarelin; JMV, rats treated with cisplatin and JMV2894.

The bands enclosed in the boxes are reported in Figure 2(C).

Supplementary figure 2 (E)

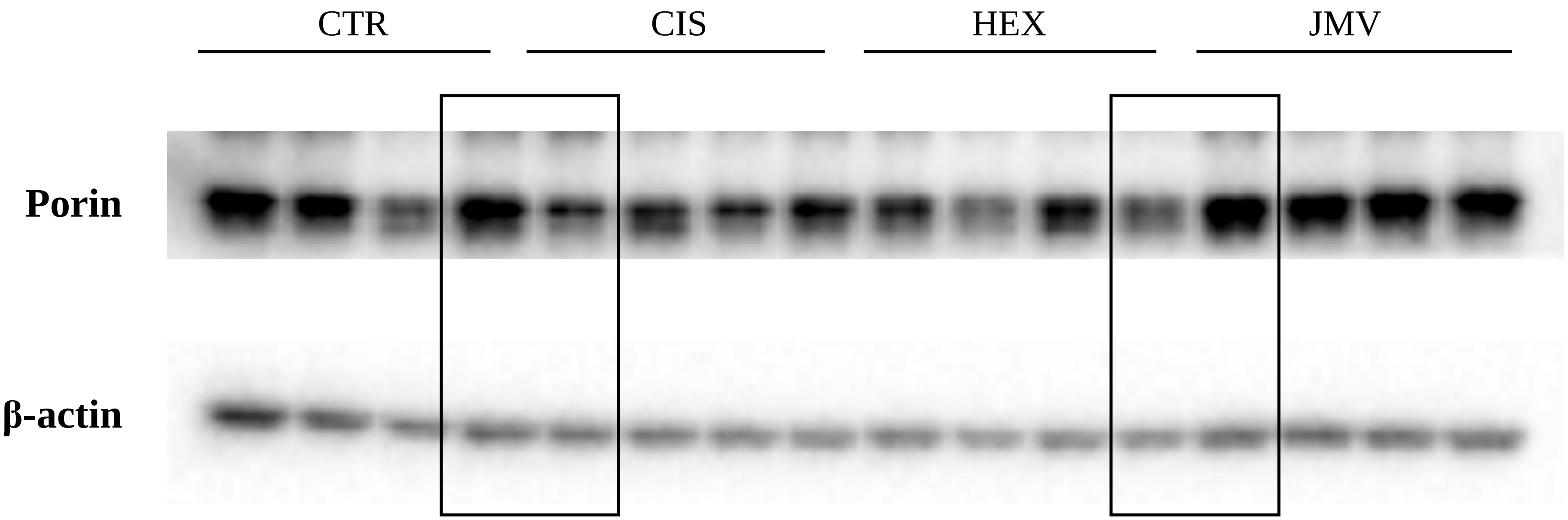

Supplementary Figure S2 (E). Representative western blotting for Porin and β-actin in tibialis anterior rat muscle. CTR, controls; CIS, rats treated with cisplatin; HEX, rats treated with cisplatin and Hexarelin; JMV, rats treated with cisplatin and JMV2894.
The bands enclosed in the boxes are reported in Figure 2(D).

Supplementary figure 2 (F)

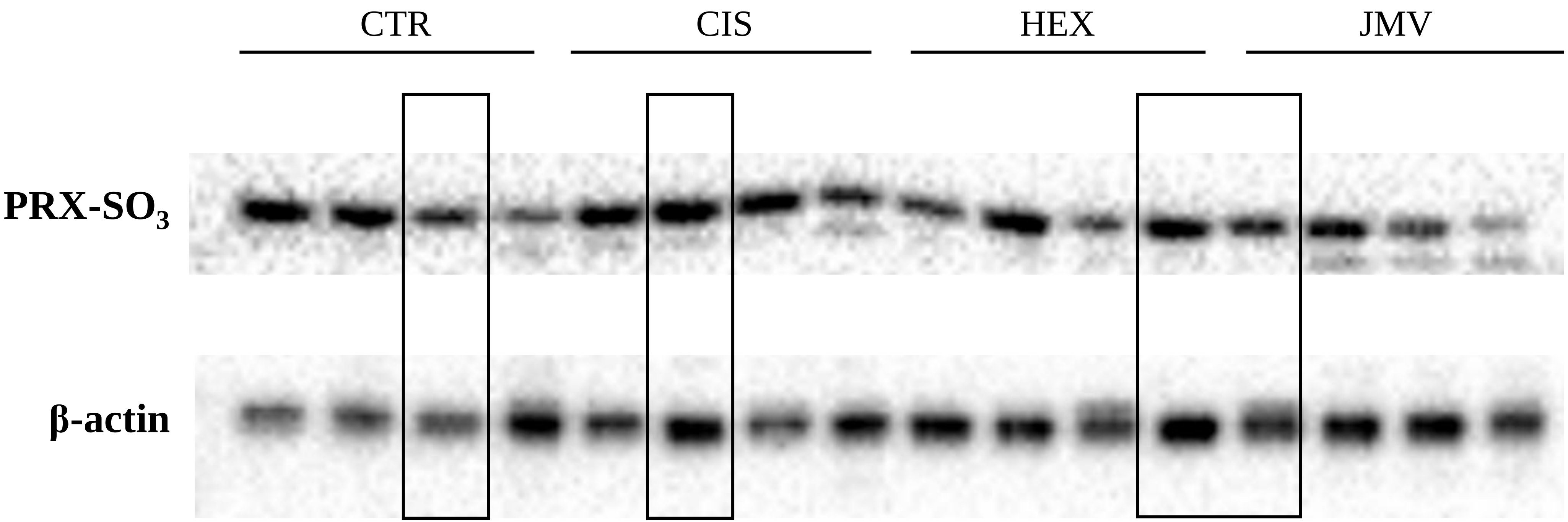

Supplementary Figure S2 (F). Representative western blotting for PRX-SO_3 and β-actin in tibialis anterior rat muscle. CTR, controls; CIS, rats treated with cisplatin; HEX, rats treated with cisplatin and Hexarelin; JMV, rats treated with cisplatin and JMV2894.

The bands enclosed in the boxes are reported in Figure 3(A).

Supplementary figure 2 (G)

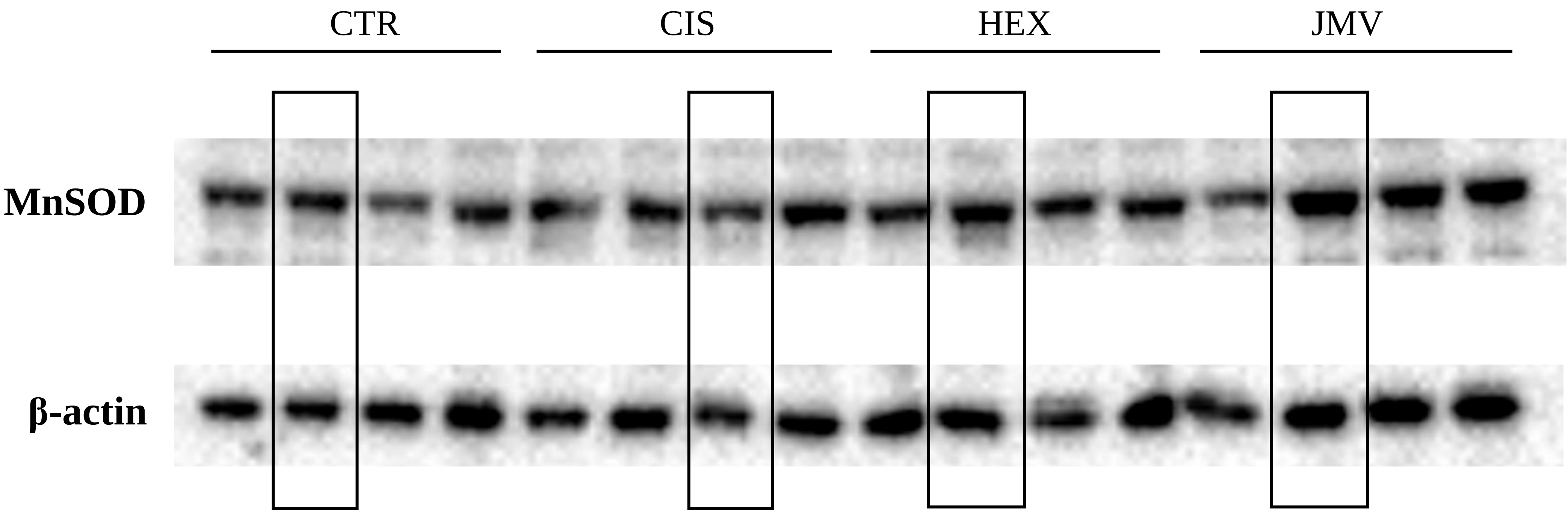

Supplementary Figure S2 (G). Representative western blotting for MnSOD and β-actin in tibialis anterior rat muscle. CTR, controls; CIS, rats treated with cisplatin; HEX, rats treated with cisplatin and Hexarelin; JMV, rats treated with cisplatin and JMV2894.
The bands enclosed in the boxes are reported in Figure 3(B).

Supplementary figure 2 (H)

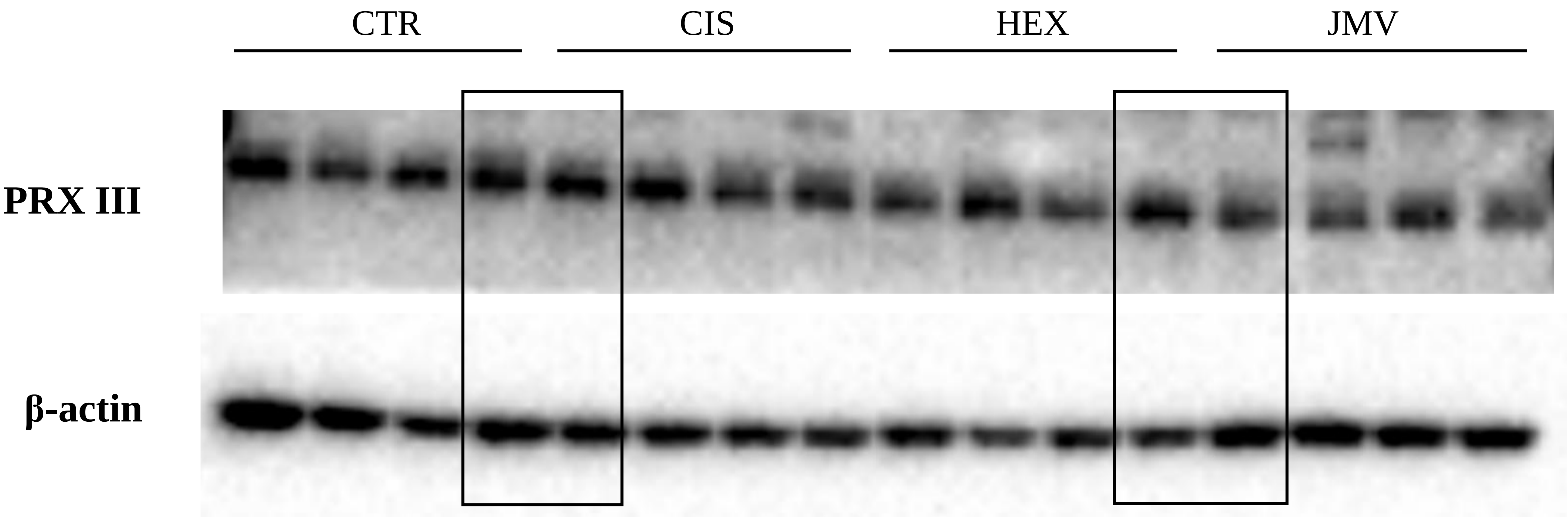

Supplementary Figure S2 (H). Representative western blotting for PRX III and β-actin in tibialis anterior rat muscle. CTR, controls; CIS, rats treated with cisplatin; HEX, rats treated with cisplatin and Hexarelin; JMV, rats treated with cisplatin and JMV2894.
The bands enclosed in the boxes are reported in Figure 3(C).

Supplementary figure 2 (I)

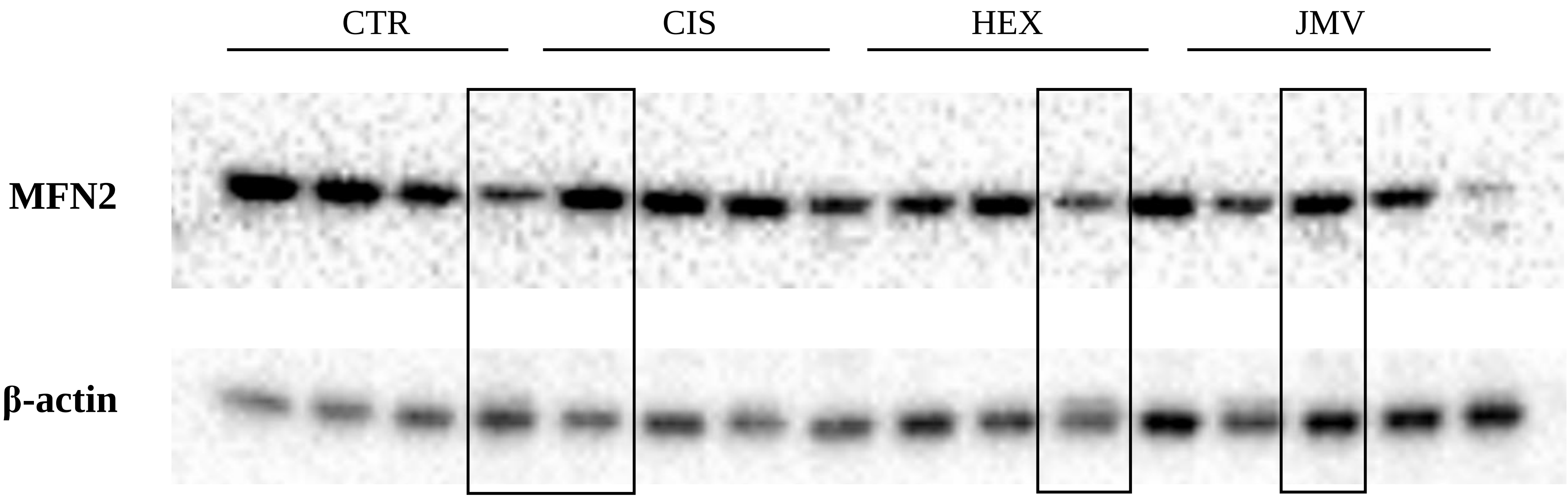

Supplementary Figure S2 (I). Representative western blotting for MFN2 and β-actin in tibialis anterior rat muscle. CTR, controls; CIS, rats treated with cisplatin; HEX, rats treated with cisplatin and Hexarelin; JMV, rats treated with cisplatin and JMV2894.
The bands enclosed in the boxes are reported in Figure 4(A).

Supplementary figure 2 (J)

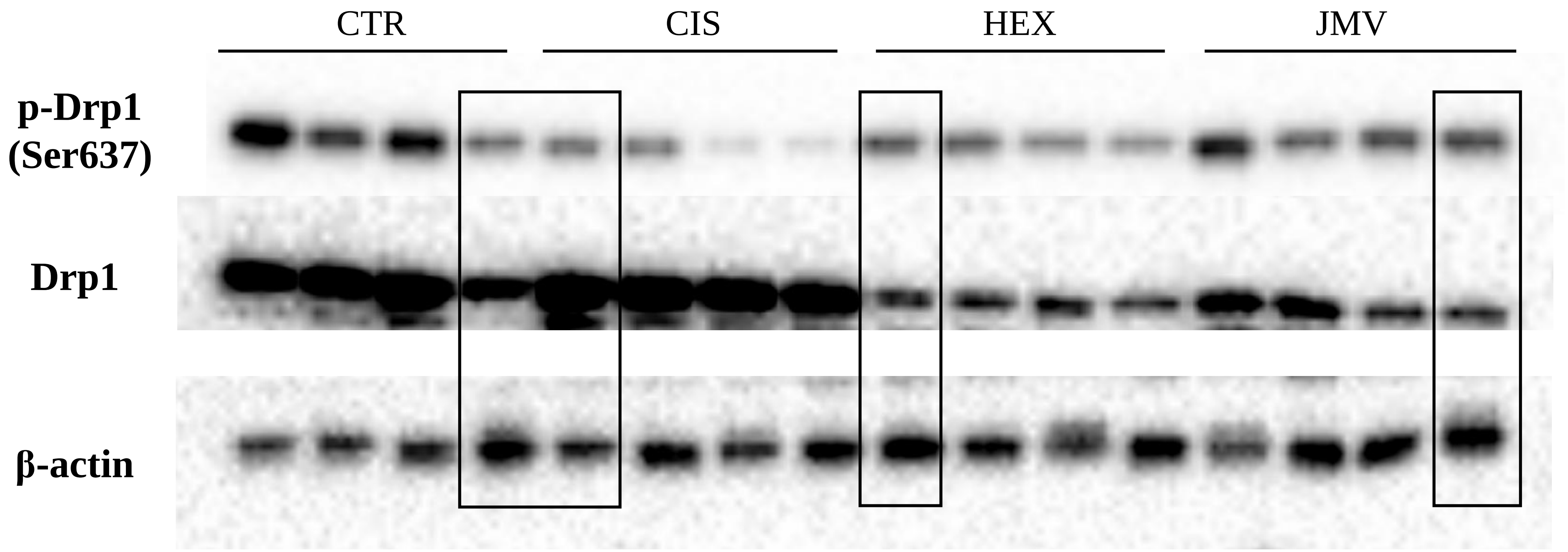

Supplementary Figure S2 (J). Representative western blotting for p-Drp1(Ser637), Drp1 and β-actin in tibialis anterior rat muscle. CTR, controls; CIS, rats treated with cisplatin; HEX, rats treated with cisplatin and Hexarelin; JMV, rats treated with cisplatin and JMV2894. The bands enclosed in the boxes are reported in Figure 4(B).

Supplementary figure 2 (K)

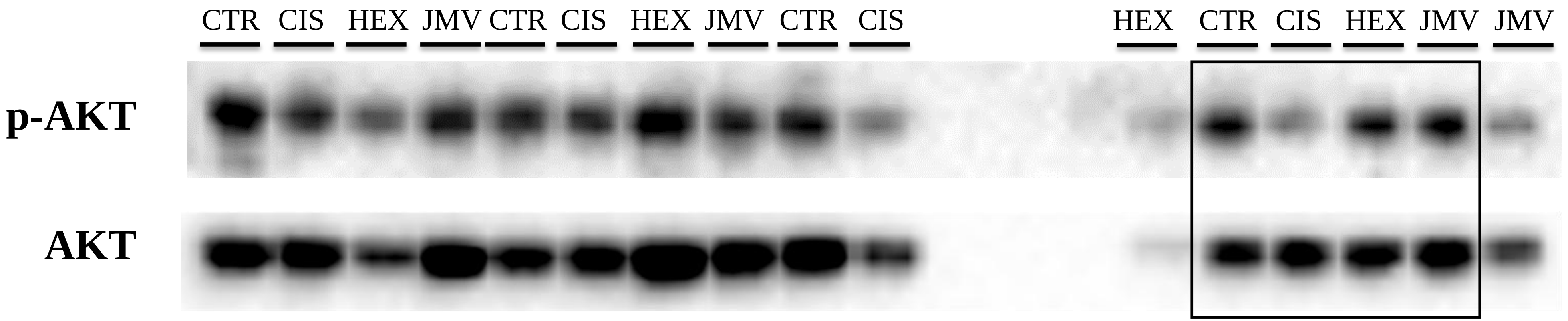

Supplementary Figure S2 (K). Representative western blotting for p-AKT and AKT in tibialis anterior rat muscle. CTR, controls; CIS, rats treated with cisplatin; HEX, rats treated with cisplatin and Hexarelin; JMV, rats treated with cisplatin and JMV2894.

The bands enclosed in the boxes are reported in Figure 5(A).

Supplementary figure 2 (L)

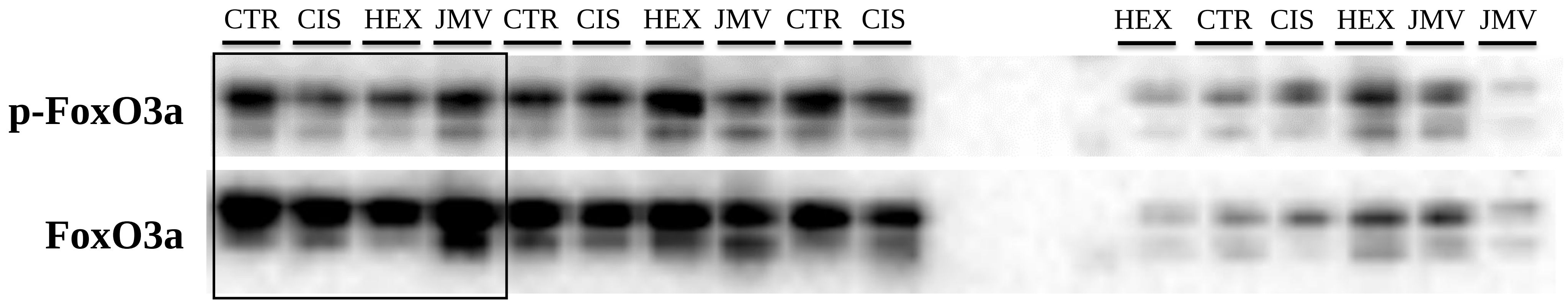

Supplementary Figure S2 (L). Representative western blotting for p-FoxO3a and FoxO3a in tibialis anterior rat muscle. CTR, controls; CIS, rats treated with cisplatin; HEX, rats treated with cisplatin and Hexarelin; JMV, rats treated with cisplatin and JMV2894.

The bands enclosed in the boxes are reported in Figure 5(B).

Supplementary figure 2 (M)

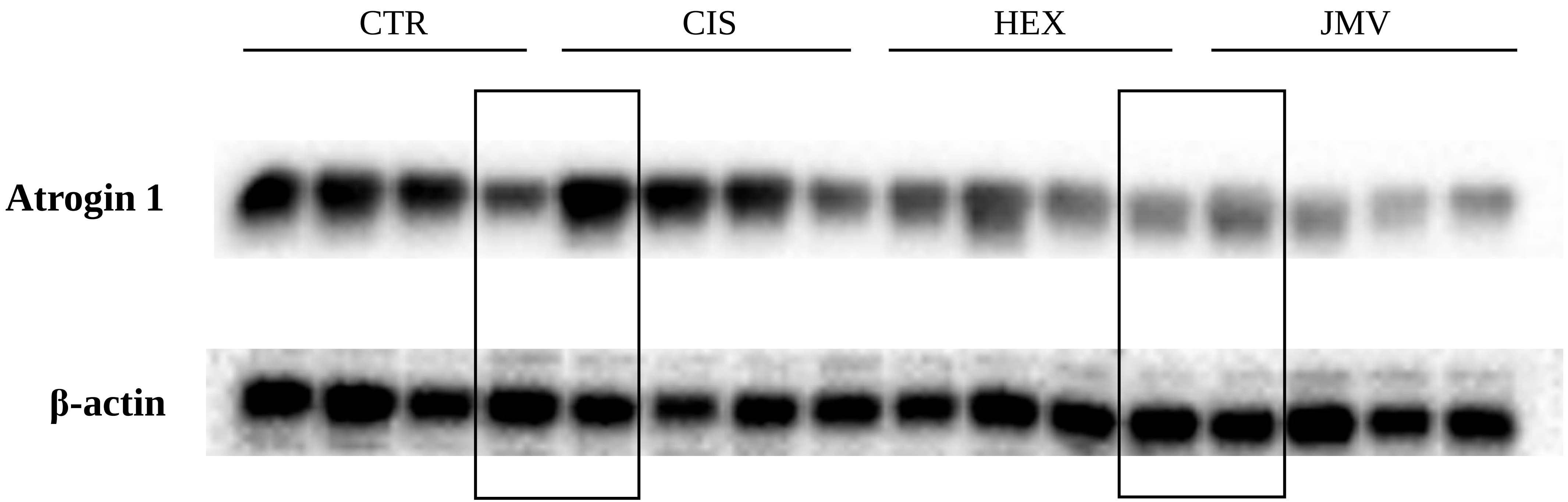

Supplementary Figure S2 (M). Representative western blotting for Atrogin 1 and β-actin in tibialis anterior rat muscle. CTR, controls; CIS, rats treated with cisplatin; HEX, rats treated with cisplatin and Hexarelin; JMV, rats treated with cisplatin and JMV2894.
The bands enclosed in the boxes are reported in Figure 6(A).

Supplementary figure 2 (N)

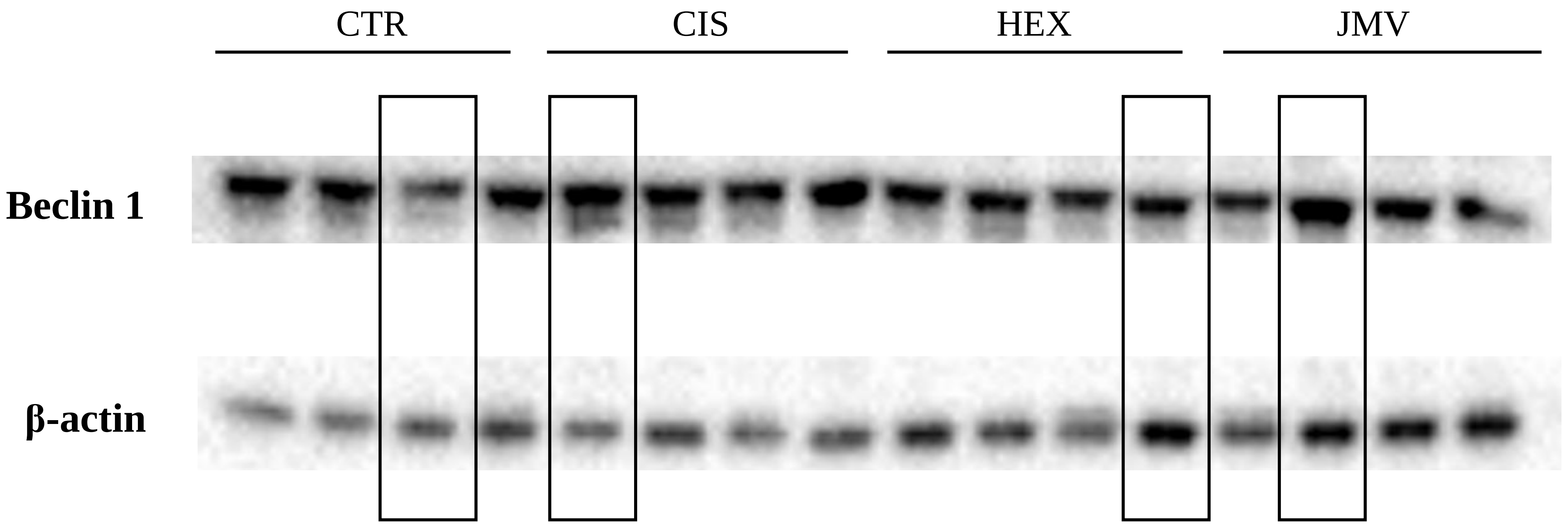

Supplementary Figure S2 (N). Representative western blotting for Beclin 1 and β-actin in tibialis anterior rat muscle. CTR, controls; CIS, rats treated with cisplatin; HEX, rats treated with cisplatin and Hexarelin; JMV, rats treated with cisplatin and JMV2894.

The bands enclosed in the boxes are reported in Figure 6(B).

Supplementary figure 2 (O)

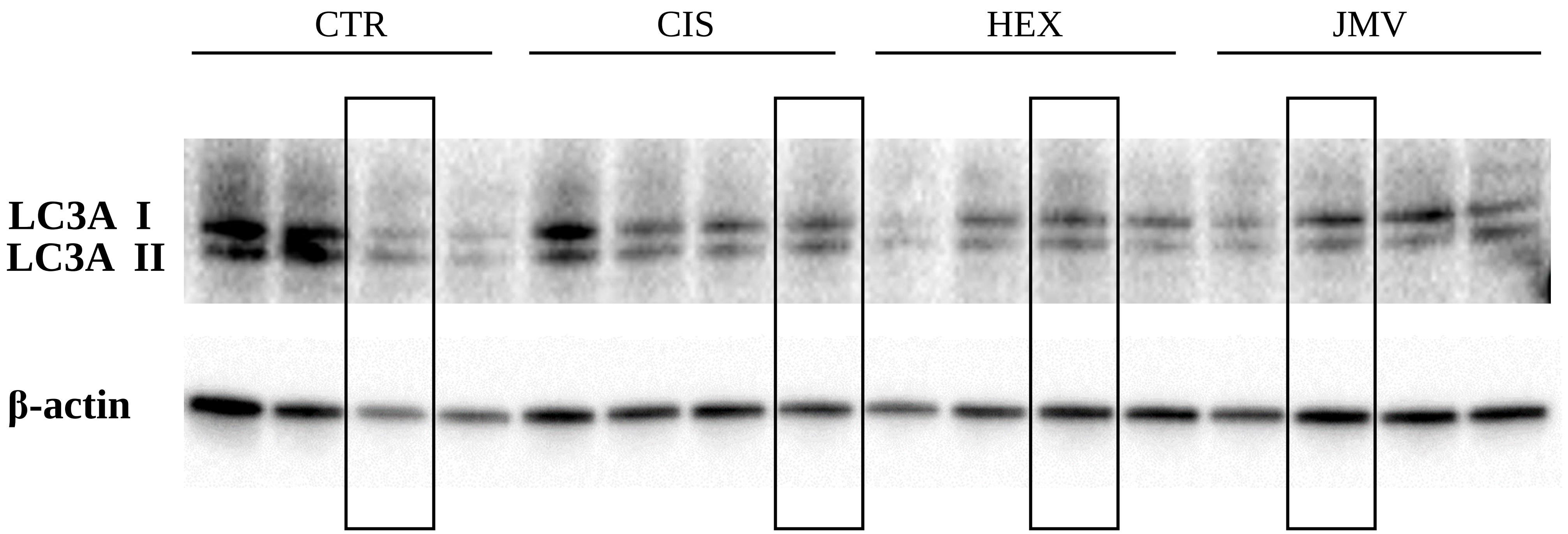

Supplementary Figure S2 (O). Representative western blotting for LC3A I, LC3A II and β-actin in tibialis anterior rat muscle. CTR, controls; CIS, rats treated with cisplatin; HEX, rats treated with cisplatin and Hexarelin; JMV, rats treated with cisplatin and JMV2894.
The bands enclosed in the boxes are reported in Figure 6(C).

Supplementary figure 2 (P)

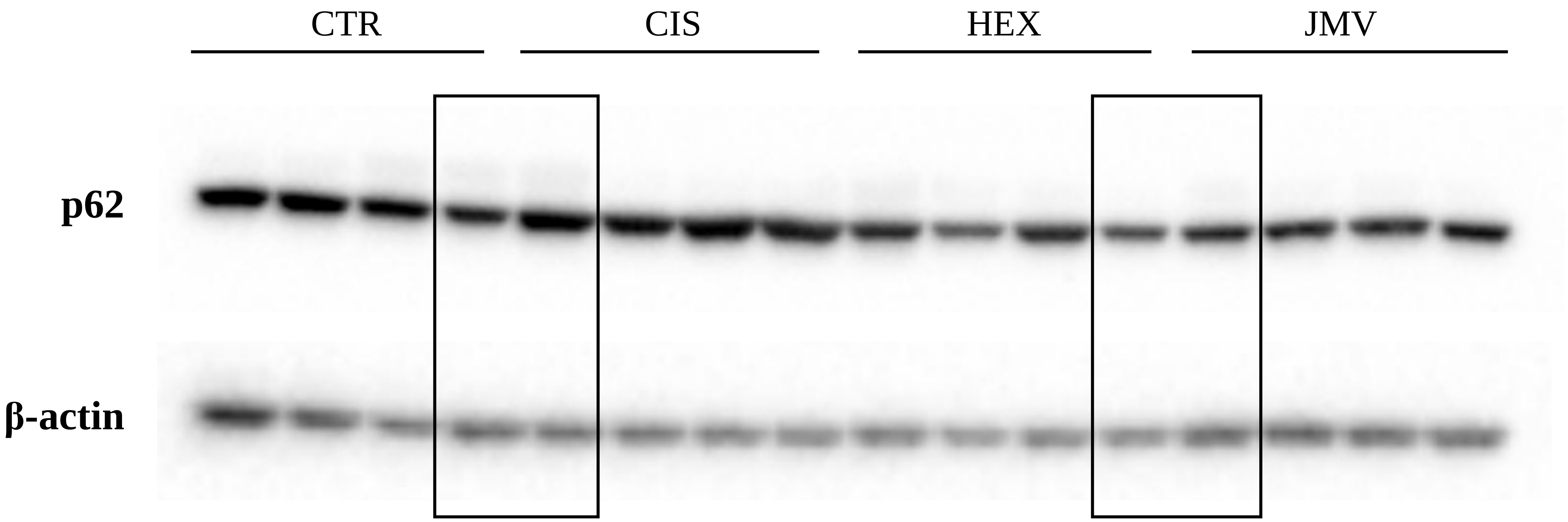

Supplementary Figure S2 (P). Representative western blotting for p621 and β-actin in tibialis anterior rat muscle. CTR, controls; CIS, rats treated with cisplatin; HEX, rats treated with cisplatin and Hexarelin; JMV, rats treated with cisplatin and JMV2894.
The bands enclosed in the boxes are reported in Figure 6(D).
